# Correlation Between Genotype and Age of Onset in Leukoencephalopathy With Vanishing White Matter

**DOI:** 10.3389/fgene.2021.729777

**Published:** 2021-10-20

**Authors:** Jiong Deng, Ling Zhou, Jie Zhang, Xuting Chang, Yuwu Jiang, Jingmin Wang, Ye Wu

**Affiliations:** Department of Pediatrics, Peking University First Hospital, Beijing, China

**Keywords:** leukoencephalopathy with vanishing white matter, EIF2B1-5, genotype, age of onset, correlation

## Abstract

**Purpose:** Leukoencephalopathy with vanishing white matter (VWM) is an autosomal recessive leukoencephalopathy caused by mutations in any of the five genes encoding the subunits of eukaryotic translation initiation factor 2B (eIF2B). The severity of the disease varies considerably, and its genotypic-phenotypic correlation is still unclear. Age of onset is the only independent clinical predictor for VWM severity. In this study, the correlation between genotype and age at onset of patients was investigated.

**Methods:** Data were collected from patients with VWM in the available literature reports and from those diagnosed in Peking University First Hospital. The age of onset was divided into early-onset (≤4 years) and late-onset type (>4 years) for the analysis of the correlation between genotype and age of onset in patients with VWM.

**Results:** A total of 341 patients were included, 281 were reported in 87 available articles and 60 were diagnosed in our center. A total of 180 different mutations were found, among which 86.1% were missense. The gene (*EIF2B1-5*) in which the mutation located, and the number of null alleles were not associated with age of onset in these patients. Certain mutations such as eIF2Bε[Arg195His] and eIF2Bε[Arg269Gln] that were predicted to have a serious influence on eIF2B structure were related to earlier age of onset. EIF2Bγ[Ala87Val] which was predicted to have a minimal influence on eIF2B structure, was related to later age of onset. Whereas eIF2Bβ[Glu213Gly], eIF2Bβ[Gly200Val] and eIF2Bε[Thr91Ala], also predicted having a small effect on the structure of eIF2B, did not show correlation with the age of onset. The onset age of patients with one or biallelic missense mutations located in the catalytic domain or other homologous domains in catalytic subunits (eIF2Bγ, ε) was earlier than that of patients with biallelic mutations located in the NT domain.

**Conclusion:** The onset age of patients with different genotypes varied greatly. The degree of influence in protein structure of some missense mutations was correlated with phenotypic severity, but the results were not completely consistent. The combined effect of biallelic mutations, the role of regulatory genes, environmental stress and other potential factors on phenotypes need to be further explored.

## Introduction

**FIGURE 1:**
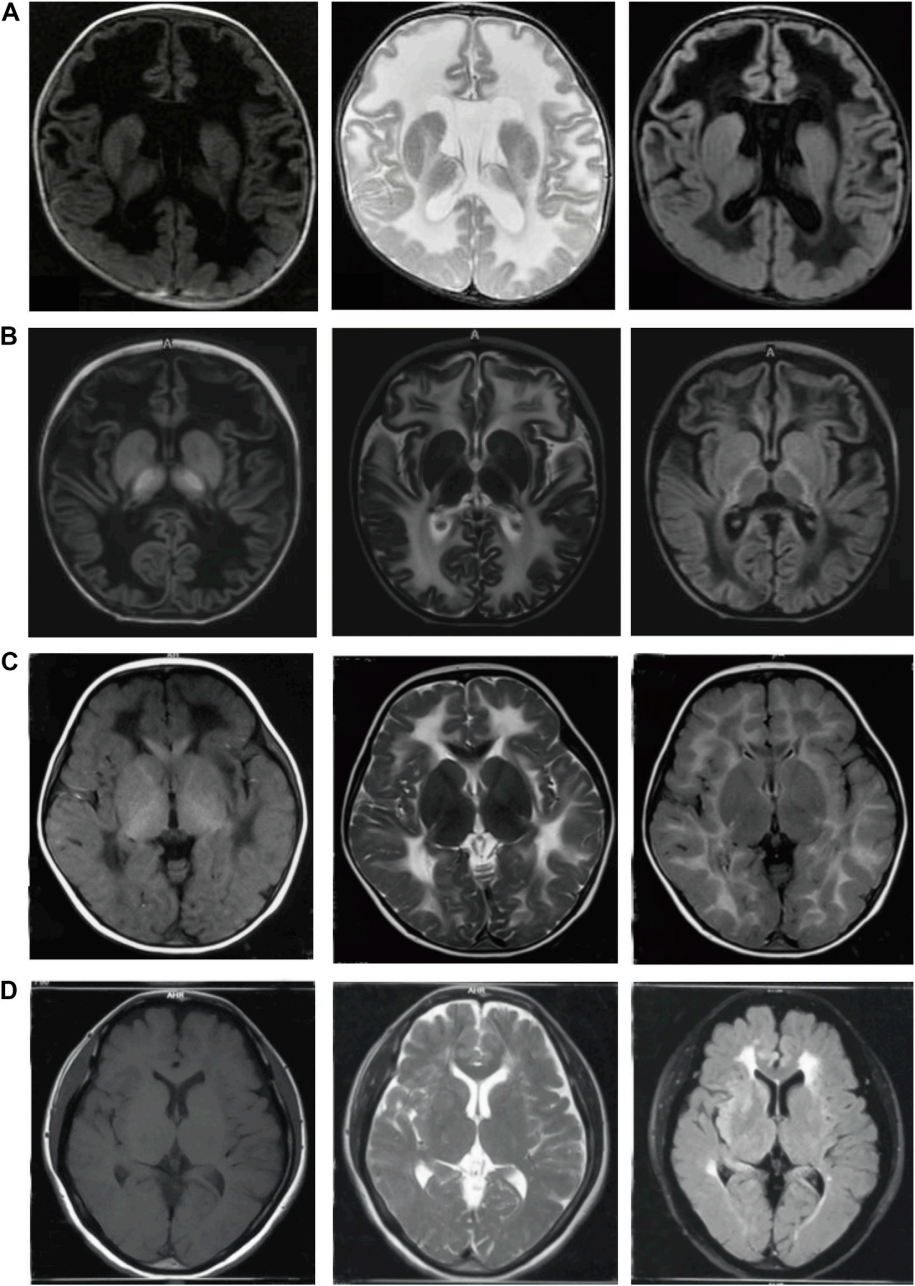
Brain MRI of four patients with VWM at different onset ages. **(A)**: case 1 with onset in neonatal period, the age at MRI was 3 months; **(B)**: case 2 with onset in infancy, the age at onset was 11 months, the age at MRI was 18 months; **(C)**: case 3 with onset in childhood, the age of onset was 3 years old, the age at MRI was 3 years old; **(D)**: case 4 with onset in adulthood, the age of onset was 43 years old, the age at MRI was 43 years old. The left column:T1 weighted sequences (T1WI); The middle column:T2 weighted sequences (T2WI); The right column: T2 FLAIR sequences. The white matter showed low signal on T1WI, high signal on T2WI and low signal liquefaction on T2 FLAIR. The earlier the age of onset, the wider the range of white matter liquefaction, while the liquefaction sign was not evident in adult patients. Furthermore, the subcortical white matter was involved in the early stage.

Leukoencephalopathy with vanishing white matter (VWM) is an autosomal recessive leukoencephalopathy. In 1997, van der Van Der Knaap et al. (1997) reported the first clinically diagnosed case of VWM. From 2001 to 2002, it was revealed to be caused by mutations in any one of the five following subunits of the eukaryotic translation initiation factor 2B(eIF2B): *EIF2B*1(12q24), *EIF2B*2(14q24), *EIF2B*3(1p34), *EIF2B*4(2p23) and *EIF2B*5(3q27) (Leegwater et al., 2001; Van Der Knaap et al., 2002). The typical phenotype in children is progressive regression in motor function exacerbated by fever or head trauma. Brain MRI shows that the white matter liquefies progressively (Figure 1). Age of onset and disease severity vary substantially from neonatal period to adulthood. Early onset of this disease is usually correlated to fast progression and poor prognosis (Hamilton et al., 2018). Although the pathogenesis of VWM is increasingly recognized, the reasons for the wide phenotype are still unknown. A clear genotype-phenotype correlation has not been established. The relationship between genotype and phenotype is important in elucidating the pathogenesis of VWM, predicting the prognosis of VWM, and promoting genetic consultation. Previous studies have confirmed that age of onset is the only independent clinical predictor of disease severity (Fogli et al., 2004a; Hamilton et al., 2018). A thorough search on patients from published literatures and those diagnosed in our center (Pediatric Department, Peking University First Hospital) were conducted to analyze the correlation between the genotype and the age of onset.

## Patients and Methods

### Inclusion of Patients

#### Literature Searching Strategy

Case series studies or case reports on VWM containing information on the clinical and genotypic spectra of patients were searched from the following databases: PubMed, Embase, Cochrane Library, Web of Science, China National Knowledge Infrastructure, and WAN FANG DATA (the latter two are Chinese public searching databases). The following search terms were used: leukoencephalopathy with vanishing white matter, vanishing white matter disease, childhood ataxia with central nervous system hypomyelination or Cree leukoencephalopathy (CLE). The publications were in either English or Chinese. The searching deadline was January 2021.

#### Inclusion Criteria of Patients From Our Center

The inclusion criteria were as follows: brain MRI showing symmetric abnormal signals on T1WI, T2WI and T2 FLAIR images in the white matter that was partially rarefied, and biallelic pathogenic or likely pathogenic variants in *EIF2B1-5* according to the American College of Medical Genetics Guidelines confirmed by sequencing (Richards et al., 2015). The study was approved by the Medical Ethics Committee of Peking University First Hospital. Informed consents were obtained from the parents of the children.

### Correlation Analysis of Genotype and Age of Onset

Age of onset was divided into early-onset type (≤4 years) and late-onset type (>4 years). It was used as a typical phenotypic index to analyze the genotype-phenotype correlation. All data were analyzed using SPSS 20.0 software. *p* < 0.05 was considered statistically significant.

#### Correlation Between the Mutant Gene (*EIF2B1-5*) and Age of Onset

Enumeration data were expressed as frequency and percentage, and measurement data were expressed as median (range). Chi-square test was performed to compare the difference in mutant genes between the early- and late-onset patients. If the data were normally distributed, one-way ANOVA was used; otherwise, Kruskal-Wallis test was applied.

#### Correlation Between Numbers of Null Allele and Age of Onset

Null allele was defined as loss-of-function allele, which included nonsense, frameshift, +1 or +2 splicing mutations, −1 and −2 intronic mutations, and single or multiple exon deletion. They were divided into 1 and 0 null allele in accordance with the number of null alleles carried by the patients. If the data were normally distributed, T test was used to analyze the age of onset between the two groups; otherwise, non-parametric test was applied.

#### Correlation Between Missense Mutations With Reported Structural Effects and Age of Onset

Some missense mutations were reported to have multiple effects on eIF2B structure, but the variant was not completely related to the phenotype, thus suggesting the influence of another allele or other modifiers on the phenotype. In this study, patients with biallelic missense variants were included for the analysis. The differences in age of onset among patients with 0, 1 or 2 missense mutations reported to show structural effects were analyzed.

#### Correlation Between Missense Mutations Located Indifferent Structural Domain and Age of Onset

**FIGURE 2:**
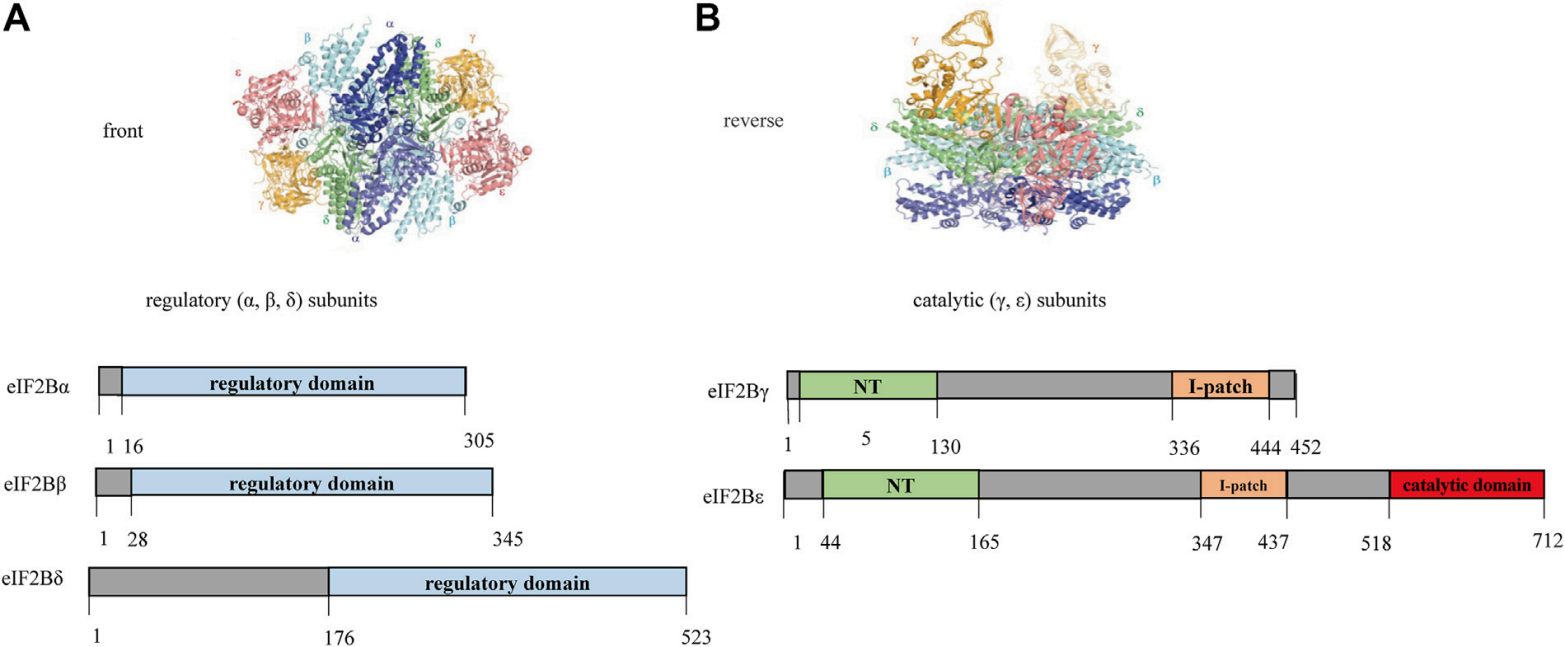
Schematic diagram of the homologous structure of human *eIF2B* (Kashiwagi et al., 2016). The eIF2B decamer is a heterodimer structure composed of regulatory (α, β, δ) and catalytic (γ, ε) subunits. The γ and ε subunits have two homology domains, the first is NT domain which sequence similarities with nucleotidyl transferases (shown in green in 5–130 amino acids of elF2Bγ and 44–165 amino acids of elF2Bε); the second is I-patch domain which has sequence similarities with acyl transferases (shown in yellow in 336–444 amino acids of elF2Bγ and 347–437 amino acids of elF2Bε), which have approximate hexad spacing of the hydrophobic branched-chain amino acids isoleucine (Ile), valine (Val), and leucine (Leu). The catalytic domain is located at the C-terminus of eIF2Bε(shown in red in 518–712 amino acids of eIF2Bε).

The eIF2B decamer consists of regulatory (α, β, δ) and catalytic (γ, ε) subunits. Catalytic subcomplexes elF2Bγ and elF2Bε have four different domains (Figure 2): 1) NT domain; 2) I-patch domain: the NT domain and I-patch domain alone do not interact with other subunits, but certain mutations or disruptions within them affected the formation of eIF2B holo-complexes; 3) Catalytic domain: responsible for the interaction of eIF2B with elF2 (the substrate); 4) other domains: except for the above three domains, the sequences are all highly homologous regions, but functional significance is still unclear. Given the wide range of effects of null allele on eIF2B and the unclear effects of intron mutations, the correlation between the affected structural domain and age of onset was compared among patients with biallelic missense variants. If the data were normally distributed, ANOVA was used to analyze the age of onset between groups affected by different structural domains; otherwise, Kruskal-Wallis test was applied.

## Results

### General Information of the Mutations

A total of 341 patients were included, among which, 281 were reported in 87 available articles and 60 were diagnosed in our center (Supplementary Table S1). In addition, 196 patients belonged to the early-onset type and 145 patients were categorized as late-onset type. The proportions of patients with mutations in *EIF2B1, EIF2B2, EIF2B3, EIF2B4,* and *EIF2B5* were 1.5% (5/341), 15.2% (52/341), 11.1% (38/341), 10.3% (35/341) and 61.9% (211/341), respectively. A total of 180 mutations were identified, including 154 missense (86.1%), 11 frameshift (6.1%), eight nonsense (4.4%), three in-frame small deletion (1.7%), 2 splice-site, and 2 intronic mutations (not splice-site).

### Correlation Between the Mutant Gene and Age of Onset

**FIGURE 3:**
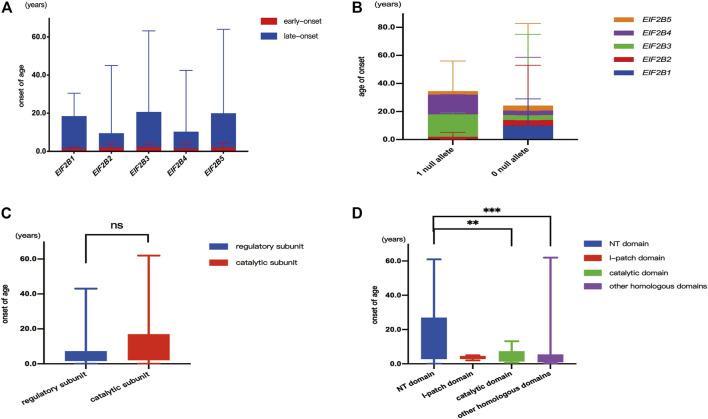
Genotype-age of onset correlation. **(A)**: Correlation between the mutant gene (*EIF2B*1-5) and age of onset. **(B)**: Correlation between number of null alleles and age of onset. **(C)**: Correlation between missense mutations located in different subunit and age at onset. **(D)**: Correlation between missense mutations located in different structural domain and age of onset. “**”denotes *p* < 0.01, “***” denotes *p* < 0.001, “ns” indicates no statistical difference.

Five patients carried mutations in *EIF2B1*, of which two were early-onset and three were late-onset, and the median age of onset was 10.0 (0.7–29.0) years. Fifty-two patients carried mutation in *EIF2B2*, of which 31 were early-onset and 21 were late-onset, and the median age of onset was 3.8 (0.2–43.0) years. Thirty-eight patients carried mutation in *EIF2B3*, of which 24 were early-onset and 14 were late-onset, and the median age of onset was 3.6 (0–61.0) years. Thirty-five patients carried mutation in *EIF2B4*, of which 21 were early-onset and 14 were late-onset, and the median age of onset was 3.5 (0–41.0) years. A total of 211 cases carried mutation in *EIF2B5*, of which 119 were early-onset and 92 were late-onset, and the median age of onset was 3.5 (0–62.0) years. No statistical difference was observed between the proportions of patients with early-onset and late-onset types among different mutant genes (χ*2* = 1.51, *p* > 0.05). No statistical difference also was observed in the age of onset among the five mutant genes (*H* = 3.39, *p* > 0.05) (Figure 3A).

### Correlation Between Numbers of Null Allele and Age of Onset

Among the study population, 36 patients carried one null allele with the median age at onset of 2.5 (0–24.0) years, and 303 patients carried zero null allele with the median age at onset of 3.6 (0–62.0) years (two patients carried intronic variants in non-splicing sites were excluded). No statistical difference in the age of onset was observed between patients with one or zero null allele (*Z* = 1.57, *p* > 0.05) (Figure 3B **)**.

### Correlation Between Missense Mutations With Reported Structural Effects and Age of Onset

Some missense mutations were predicted to show variable structural effects on the protein, including eIF2Bβ[Thr91Bla], eIF2Bβ[Ser171Phe], eIF2Bβ[Gly200Val], eIF2Bγ [Arg91His], eIF2Bδ [Arg264Trp], eIF2Bδ[Arg357Trp], eIF2Bδ[Arg483Trp], eIF2Bε [Thr79Ile], eIF2Bε[Glu81Leu], eIF2Bε[Arg136His], eIF2Bε[Arg195His], eIF2Bε[His269Pro], eIF2Bε[Arg316Gln], eIF2Bε[Thr91Ala], eIF2Bε[Glu213Gly], eIF2Bε[Arg133His], eIF2Bε[Gly386Val], [Ala403Val] and [Thr432Ile](Slynko et al., 2021).

In this study, due to the small number of the above mutations in these patients, we only analyzed eIF2Bε[Arg195His], eIF2Bε[Arg269Gln], eIF2Bβ[Gly200Val], eIF2Bβ[Glu213Gly], eIF2Bγ[Ala87Val] and eIF2Bε[Thr91Ala]. Among them, eIF2Bε[Arg195His] and eIF2Bε[Arg269Gln] were considered to have a serious effect on the structure of eIF2B and associated with a more serious phenotype, whereas eIF2Bβ[Gly200Val], eIF2Bβ[Glu213Gly], eIF2Bγ[Ala87Val] and eIF2B[Thr91Ala] were predicted to have a small effect on the structure of eIF2B and associated with a mild phenotype. Only patients with biallelic missense mutations were included for the analysis.

Differences in the age of onset among patients with 0, 1 or 2 above missense mutations were analyzed (Table 1). The age of onset in patients with eIF2Bε[Arg195His] or eIF2Bε[Arg269Gln] mutation was significantly earlier than that in patients without the mutations (*Z* = 2.14, *p* < 0.05; *Z* = 2.77, *p* < 0.01), thus supporting the previous results that patients with these two mutations have a more serious clinical phenotype. In addition, the patients with two eIF2Bε[Arg195His] variants had an earlier age of onset than those with one or no eIF2Bε[Arg195His], and patients with one eIF2Bε[Arg195His] showed no statistically significant difference in the age of onset with those without the mutation. Similarly, the patients with one eIF2Bε[Arg269Gln] had an earlier age of onset than those without the mutation. The age at onset in patients with eIF2Bγ[Ala87Val] or eIF2Bγ[Arg269Gln] mutation was significantly later than that in patients without the mutation (*Z* = 2.68, *p* < 0.01), indicating that the slight structural effect predicted mild disease severity. However, no significant difference in the age of onset was observed among patients with eIF2Bβ[Gly200Val], eIF2Bβ[Glu213Gly] or eIF2Bε [Thr91Ala] compared with other patients (*Z* = 1.34, *p* > 0.05; *Z* = 0.94, *p* > 0.05; *Z* = 0.11, *p* > 0.05, respectively), suggesting that eIF2Bβ[Glu213Gly], eIF2Bβ[Gly200Val] and eIF2Bε[Thr91Ala] have a slight correlation with age of onset in these patients.

**TABLE 1:** Correlation between age of onset among patients with 0, 1 or 2 above missense mutations and structural effect.

Predicted structural effects	Mutations	Numbers of mutations	*N* ^a^	Age of onset(years)	Variable	*P*
substantial effect	eIF2Bε[Arg195His]	1–2	13	0.8(0.3–34)	13.08	<0.001
		2	6	0.7(0.3–0.9)	H_a_ = 3.63	<0.001
					H_b_ = 0.09	>0.05
		1	7	4.5(0.3–34.0)	H_c_ = 0.41	>0.05
		0	290	3.6(0–62.0)		
substantial effect	eIF2Bε[Arg269Gln]	1–2	9	1.2(0.7–4.5)	2.77	<0.01
		2	2	(1.2–1.6)		
		1	7	1.1(0.7–4.5)	H_d_ = 2.34	<0.05
		0	294	3.7(0–62.0)		
small effect	eIF2Bβ[Glu213Gly]	1–2	22	5.5(1.5–17.0)	1.84	>0.05
		2	17	5.0(1.5–17.0)		
		1	5	7.0(1.7–17.0)		
		0	281	3.5(0–62.0)		
small effect	eIF2Bβ[Gly200Val]	1	7	1.7(0.3–22.0)	0.94	>0.05
		0	296	3.6(0–62.0)		
small effect	eIF2Bε[Thr91Ala]	1–2	5	2.0(1.5–3.0)	0.11	>0.05
		0	298	3.6(0–62.0)		
small effect	eIF2Bγ[Ala87Val]	1–2	10	29.0(2.1–61.0)	2.68	<0.01
		2	6	26.5(2.1–61.0)		
		1	4	38.5(2.3–57.0)		
		0	293	3.5(0–62.0)		

a *N*: number of patients with 0, 1 or 2 missense mutations; *H*_*a*_: people carried two eIF2Bε[Arg195His] compared with those without eIF2Bε[Arg195His] mutation; *H*_*b*_: people carried two eIF2Bε[Arg195His] compared with those carried one eIF2Bε[Arg195His] mutation; *H*_*c*_: people carried one eIF2Bε[Arg195His] compared with those without eIF2Bε[Arg195His] mutation. *H*_*d*_: people carried one eIF2Bε[Arg269Gln] compared with those without eIF2Bε[Arg269Gln] mutation.

### Correlation Between Missense Mutations Located in Different Structural Domain and Age of Onset

Among the 154 missense mutations, four were *EIF2B*1 mutations, 17 were *EIF2B*2 mutations, 23 were *EIF2B*3 mutations, 28 were *EIF2B*4 mutations, and 82 were *EIF2B*5 mutations (Supplementary Table S2).

#### Correlation Between Missense Mutations Located in Different Subunit and Age at Onset

Among the 303 patients carrying biallelic missense mutations, 81 patients carried mutations affecting the regulatory subunit, and the median age of onset was 4.0 (0–43.0) years, while 222 patients carried mutations affecting the catalytic subunit, and the median age of onset was 3.5 (0–62.0) years. No statistically significant difference in the age of onset was observed among patients carrying mutations affecting the regulatory or catalytic submit (*Z* = 1.26, *p* > 0.05) (Figure 3C).

#### Correlation Between Missense Mutations Located in Different Structural Domain and Age of Onset

A total of 222 patients carrying missense mutations in the catalytic subunit were classified into the following four groups: 1) 106 patients carried biallelic mutations in the NT domain, with the median age of onset 9.0 (0–61.0) years; 2) five patients carried biallelic mutations in the I-patch domain, with the median age of onset 4.0 (2.0–5.0) years; 3) eight patients carried one mutation affected the catalytic domain, with the median age of onset 2.5 (0.7–13.2) years; and 4) 49 patients carried biallelic mutations affecting the other domains, with the median age of onset 2.5 (0.3–62.0) years. Significant difference in the age of onset was observed among these four groups (*H* = 19.19, *p* < 0.001). The age of onset in patients carrying one mutation in the catalytic domain was significantly earlier than that of patients with two mutations in the NT domain (*Z* = 2.28, *p* < 0.05). The age of onset in patients carrying biallelic mutations affecting other domains was earlier than that in patients with biallelic mutations in the NT domain (*Z* = 3.99, *p* < 0.001) (Figure 3D).

## Discussion

A genotype-phenotype correlation was reported in patients with VWM, but certain differences were found, in part because of differences in the phenotypic measures used in the studies (Fogli et al., 2004a; Van Der Lei et al., 2010; Matsukawa et al., 2011). Age of onset is the only independent clinical predictor of disease severity (Hamilton et al., 2018), which is easily accessible from the literature, and therefore served as a relatively reliable index of phenotype in our study. Data from 341 patients with VWM were collected, including 180 different mutations in *EIF2B1-5*, of which 86.1% were missense mutations. In this study, the proportions of patients with mutations in *EIF2B1, EIF2B2, EIF2B3, EIF2B4,* and *EIF2B5* were 1.5, 15.2, 11.1, 10.3 and 61.9%, respectively. Similar to the results of previous reports (Bugiani et al., 2010), the most common mutant gene was *EIF2B5*, followed by *EIF2B2*. The correlation between the age of onset and the different mutant genes was not identified.

*EIF2B1-5* are house-keeping genes that play an important role in eukaryotic cells. They encode eIF2B, which is a critical factor in the initiation of protein translation. Null allele may result in the inability to synthesize eIF2B protein and loss of translation initiation function. Therefore, individuals carrying two null alleles could not survive to be born. No patients carrying two null alleles have been reported yet. In this study, we analyzed the correlation between numbers of null allele and age of onset. We did not find significant difference between the age of onset in patients with one or zero null allele. This finding may be due to the relatively small proportion of patients with null allele (10.5%).

Previous studies have reported a certain correlation between the degree of influence of missense mutations on protein structure and phenotypes, but the results are not consistent. Results from protein prediction software or model analysis of the mutation site function prediction revealed that some mutations with multiple or substantial effects on the eIF2B structure were associated with severe phenotypes, such as eIF2Bε[Thr79Ile], eIF2Bε[Glu81Leu], eIF2Bε[Arg136His], eIF2Bε[Arg195His], eIF2Bε[His269Pro], eIF2Bε[Arg269Gln], eIF2Bε[Arg316Gln]; eIF2Bδ[Arg264Trp], eIF2Bδ[Arg357Trp], eIF2Bδ[Arg483Trp], and eIF2Bγ[Arg91His] (Slynko et al., 2021). Mutations such as eIF2Bβ[Thr91Bla], eIF2Bε[Thr91Ala], eIF2Bε[Ala87Val] and eIF2Bε[Glu213Gly], with predicted minimal influence on the eIF2B structure, were associated with mild disease severity. However, the functional prediction of missense variants could not completely explain the phenotype. For example, mutations such as eIF2Bε[Arg133His] and eIF2Bβ[Ser171Phe] were predicted to have a huge effect on protein structure, but they were related to mild phenotype. Mutations such as eIF2Bβ[Gly200Val], eIF2Bε[Gly386Val] [Ala403Val] and [Thr432Ile], with small predict effects, were linked to serious phenotype (Fogli et al., 2004b; Campbell and Ashe, 2006; Slynko et al., 2021). In this study, 303 patients with biallelic missense mutations were analyzed. Those with eIF2Bε[Arg195His] and eIF2Bε[Arg269Gln] had a significantly earlier age of onset than those without the mutations. This finding is consistent with the genotype-phenotype correlation from previous reports. The phenotype of children carrying eIF2Bε[Arg195His] mutation usually manifested as Cree leukoencephalopathy, a rapidly fatal phenotypic variant of infantile VWM with disease onset in prenatal or infancy; almost all of the affected patients died before 2 years of age (Van Der Knaap et al., 2003; Harder et al., 2010). In the present study, patients carrying eIF2Bγ[Ala87Val] had a significantly later age of onset than those without the mutation. Contrary to the results of previous studies, eIF2Bβ[Glu213Gly], [Gly200Val], and eIF2Bε[Thr91Ala] showed a minimal correlation with the age of onset. Fogli et al. (2004a) reported that eIF2Bβ[Glu213Gly] mutation was associated with a mild phenotype, relatively late age of onset, and slow progression. van der Van Der Lei et al. (2010) found that the disease severity of patients with homozygous eIF2Bε[Arg113His] was milder than that of patients with heterogeneous eIF2Bε[Arg113His] and homozygous eIF2Bε[Thr91Ala]. Meanwhile, the phenotype of patients with eIF2Bε[Arg113His/Arg339any] was milder than that of patients with eIF2Bε[Thr91Ala/Arg339any]. In our study, the age of onset among patients with two, one or without eIF2Bε[Arg195His] variants were compared. The patients with biallelic eIF2Bε[Arg195His] showed earlier onset than those with one or no variant, and the patients with one eIF2Bε[Arg195His] showed no statistically significant difference in terms of age of onset compared with those without the variant. Similarly, the patients with one eIF2Bε[Arg269Gln] mutation had an earlier age of onset than those without the mutation. These findings suggested that the correlation between genotype and phenotype required consideration of the combined effects of biallelic variants.

Whether the location of the missense mutations in different types of subunits or functional domains was associated with the age of onset was further analyzed. The results showed that the age of onset was not correlated with the location of the mutations in the regulatory (α, β, δ) or catalytic (γ, ε) subunits. Catalytic subcomplexes elF2Bγ and elF2Bε have four different domains: NT domain, I-patch domain, catalytic domain and other domains. eIF2Bε has the most important catalytic domain (amino acids 518–712 at the C-terminus) with GEF activity (GDP/GTP conversion). Knockout of the catalytic domain fragment in the mouse model could substantially reduce GEF activity and eIF2Bε binding to other subunits, suggesting that this region is important for the stability and activity of the elF2B complex (Wortham et al., 2016; Moon and Parker, 2018a). Therefore, we further analyzed whether the location of missense mutations in different domains of the catalytic subunits influenced the age of onset. The results showed that patients carrying mutations located in the catalytic domain and other homologous domains had an earlier age of onset.

In VWM, stress is the triggering or aggravating factor, including head trauma, acute startle, and febrile infection (Labauge et al., 2009). Different cellular stresses lead to eIF2α phosphorylation, which then acts as a competitive inhibitor of eIF2B, inhibiting mRNA translation. A central mechanism in a cellular stress response is the inhibition of protein synthesis, known as unfolded protein response (Wong et al., 2019). This process aims to reduce the accumulation of denatured and misfolded proteins and escape translation inhibition through the transcription of mRNA with a special open reading frame (ORF), thus preserving cellular energy to enhance cell survival under stress. However, if the cellular stress state remains uncorrected for a long time and the unfolded protein response is overactivated and could not be recovered over a long period of time, it may lead to the logical deterioration and possible multi-organ dysfunction, which may be the possible cause of exacerbation of VWM (Moon and Parker, 2018b). Therefore, the observed considerable phenotypic variability indicated that genotype is not the only factor that determines phenotype; the environment may be an essential influential entity that affects disease severity. Exposure to factors causing episodic exacerbations could worsen the disease. Measures to prevent trauma and infection can improve the prognosis, and other factors, such as the regulation of upstream and downstream gene variation and epigenetics, have possible implications.

This study has some limitations. Most mutations have not been functionally studied, and their effects on protein functions need to be confirmed. The variants of only one allele were analyzed, and the other allele also contributed to the phenotype. The combined effect of the two alleles requires a larger sample data. Other regulatory genes and environmental factors also influence phenotypes, and they were not considered in this study. Age of onset is an independent risk factor for phenotypic severity. It was used as a phenotypic factor in this study to simplify and facilitate the extraction from the literature. However, it was likely to be inaccurate, and including indicators, such as the speed of motor function progression, is preferable.

## Conclusion

A detailed genotypic-phenotypic association analysis was performed in 341 patients with VWM. The gene (*EIF2B1-5*) in which the mutation located, and the number of null alleles were not associated with age of onset in these patients. Certain mutations, such as eIF2Bε[Arg195His] and eIF2Bε[Arg269Gln], which were predicted to have a serious influence on eIF2B structure, were related to earlier age of onset. And eIF2Bγ[Ala87Val], which was predicted to have a minimal influence on eIF2B structure, was related to later onset. eIF2Bβ[Glu213Gly], eIF2Bβ[Gly200Val] and eIF2Bε[Thr91Ala] showed a slight correlation with age of onset. The age of onset in patients with one or biallelic missense mutations located in the catalytic domain or other homologous domains in the catalytic subunits was earlier than that of patients with biallelic variants located in the NT domain. The combined effect of biallelic mutations, the role of regulatory genes, environmental stress and other potential factors on phenotypes need to be further explored.

## Data Availability

The original contributions presented in the study are included in the article and Supplementary Material, further inquiries can be directed to the corresponding author.
