# Supplementary material for: Correlation Between Genotype and Age of Onset in Leukoencephalopathy With Vanishing White Matter
Source: Front Genet. 2021 Oct 20;12:729777. doi: 10.3389/fgene.2021.729777 (PMC8564072; doi:10.3389/fgene.2021.729777)
Supplement: Supplementary file 1 [file Table1.DOCX]

Supplement Table 1.Overview of genotype and age of onset of 341 VWM patients

| Patient | Sex | age of onset，y | mutated gene | DNA mutation 1 | Protein change 1 | DNA mutation 2 | Protein change 2 | Reference |
| --- | --- | --- | --- | --- | --- | --- | --- | --- |
| 1 | F | 0.5 | *EIF2B5* | c.584G>A | p. Arg195His | c.436T>C | p.Ser146Pro | Robbins, Arraj et al. 2021(Robbins et al., 2021) |
| 2 | uk | antenatally | *EIF2B5* | c.468C>G | p.Ile156Met | c.1165G>A | p.Val389Met | Trimouille, Marguet et al. 2020(Trimouille et al., 2020) |
| 3 | uk | antenatally | *EIF2B5* | c.468C>G | p.Ile156Met | c.1165G>A | p.Val389Met | Trimouille, Marguet et al. 2020(Trimouille et al., 2020) |
| 4 | F | 15.0 | *EIF2B3* | c.260C>T | p.Ala87Val | c.260C>T | p.Ala87Val | Cohen, Manín et al. 2020(Cohen et al., 2020) |
| 5 | M | 0 | *EIF2B4* | c.725C>T | p.Pro242Leu | c.1301T>C | p.Leu434Pro | Bursle, Yiu et al. 2020(Bursle et al., 2020) |
| 6 | M | 5.0 | *EIF2B4* | c.628G>T | p.Gly210Cys | c.725C>T | p.Pro242Leu | Bursle, Yiu et al. 2020(Bursle et al., 2020) |
| 7 | M | 14.0 | *EIF2B5* | c.338G>A | p.Arg113His | c.338G>A | p.Arg113His | Vinogradsky and Otallah 2019(Vinogradsky and Otallah, 2019) |
| 8 | F | 1.4 | *EIF2B3* | c.706C>G | p.Gln236Glu | c.89T>C | p.Val30Ala | Hyun, Choi et al. 2019(Hyun et al., 2019) |
| 9 | F | 41.0 | *EIF2B4* | c.626G>A | p.Arg209Gln | c1298​​C>T | p.Pro433Leu | Buggle, Ciric et al. 2019(Buggle et al., 2019) |
| 10 | M | 38.0 | *EIF2B3* | c.260C>T | p.Ala87Val | c.260C>T | p.Ala87Val | Accogli, Brais et al. 2019(Accogli et al., 2019) |
| 11 | F | 18.0 | *EIF2B5* | c.338G>A | p.Arg113His | c.338G>A | p.Arg113His | Villar-Quiles, Delgado-Suarez et al. 2018(Villar-Quiles et al., 2018) |
| 12 | M | 0.4 | *EIF2B5* | c.896G>A | p.Arg299His | c.896G>A | p.Arg299His | Porciuncula, Spada et al. 2018(Porciuncula et al., 2018) |
| 13 | F | 3.0 | *EIF2B5* | c.338G>A | p.Arg113His | c.1694delAins45 | p.Lys565Ilefs*3 | Pena, Jiang et al. 2018(Pena et al., 2018) |
| 14 | F | 0.7 | *EIF2B4* | c.614C>T | p.Pro205Leu | c.614C>T | p.Pro205Leu | Hettiaracchchi, Neththikumara et al. 2018(Hettiaracchchi et al., 2018) |
| 15 | F | 1.1 | *EIF2B5* | c.1688G>A | p.Arg563Gln | c.806G>A | p.Arg269Gln | Bektas, Yesil et al. 2018(Bektas et al., 2018) |
| 16 | M | 30.0 | *EIF2B5* | c.338G>A | p.Arg113His | c.338G>A | p.Arg113His | Barros, Parreira et al. 2018(Barros et al., 2018) |
| 17 | M | 0 | *EIF2B5* | c.956A>G | p.Tyr319Cys | c.1546+1G>T | p.? | Yavuz 2017(Yavuz, 2017) |
| 18 | M | 0.2 | *EIF2B5* | c.956A>G | p.Tyr319Cys | c.1546+1G>T | p.? | Yavuz 2017(Yavuz, 2017) |
| 19 | M | 0.8 | *EIF1B5* | c.1015C>T | p.Arg339Trp | c.1208C>T | p.Ala403Val | van Diemen, Kerstjens-Frederikse et al. 2017(van Diemen et al., 2017) |
| 20 | M | antenatally | *EIF2B3* | c.97A>G | p.Lys33Glu | c.97A>G | p.Lys33Glu | Song, Haeri et al. 2017(Song et al., 2017) |
| 21 | M | antenatally | *EIF2B3* | c.97A>G | p.Lys33Glu | c.97A>G | p.Lys33Glu | Song, Haeri et al. 2017(Song et al., 2017) |
| 22 | F | 2.5 | *EIF2B5* | c.241G>A | p.Glu81Lys | c.203T>C | p.Leu68Ser | Singh, Livingston et al. 2017(Singh et al., 2017) |
| 23 | F | 4.0 | *EIF2B2* | c.254T>A | p.Val85Glu | c.677T>A | p.Met226Lys | Lee, Lee et al. 2017(Lee et al., 2017) |
| 24 | F | 12.0 | *EIF2B2* | c.254T>A | p.Val85Glu | c.677T>A | p.Met226Lys | Lee, Lee et al. 2017(Lee et al., 2017) |
| 25 | M | 3.5 | *EIF2B3* | c.1270T>G | p.Cys424Gly | c.1270T>G | p.Cys424Gly | Gowda, Srinivasan et al. 2017(Gowda et al., 2017) |
| 26 | M | 1.1 | *EIF2B5* | c.318A>T | p.Leu106Phe | c.318A>T | p.Leu106Phe | Esmer, Blanco Hernández et al. 2017(Esmer et al., 2017) |
| 27 | M | 3.5 | *EIF2B4* | c.1334G>A | p.Arg445His | c.1334G>A | p.Arg445His | Wang, He et al. 2016(Wang et al., 2016) |
| 28 | F | 1.2 | *EIF2B5* | c.806G>A | p.Arg269Gln | c.806G>A | p.Arg269Gln | Wang, He et al. 2016(Wang et al., 2016) |
| 29 | M | 2.4 | *EIF2B5* | c.1004G>C | p.Cys335Ser | c.1484A>G | p.Tyr495Cys | Wang, He et al. 2016(Wang et al., 2016) |
| 30 | M | 0.7 | *EIF2B1* | c.146T>G | p.Leu49Arg | c.146T>G | p.Leu49Arg | ALAMRI H et al.2016 (Alamri et al., 2016) |
| 31 | F | 0.3 | *EIF2B5* | c.584G>A | p.Arg195His | c.1223T>C | p.Ile408Thr | Takano, Tsuyusaki et al. 2015(Takano et al., 2015) |
| 32 | F | 29.0 | *EIF2B1* | c.715T>G | p.Phe239Val | c.715T>G | p.Phe239Val | Shimada, Shimojima et al. 2015(Shimada et al., 2015) |
| 33 | F | 3.0 | *EIF2B2* | c.254T>A | p.Val85Glu | c.254T>A | p.Val85Glu | Shimada, Shimojima et al. 2015(Shimada et al., 2015) |
| 34 | M | 0.7 | *EIF2B2* | c.254T>A | p.Val85Glu | c.682A>G | p.Arg228Gly | Shimada, Shimojima et al. 2015(Shimada et al., 2015) |
| 35 | M | 13.0 | *EIF2B4* | c.556T>A | p.Tyr186Asn | c.1070G>A | p.Arg357Gln | Shimada, Shimojima et al. 2015(Shimada et al., 2015) |
| 36 | M | 13.0 | *EIF2B4* | c.556T>A | p.Tyr186Asn | c.1070G>A | p.Arg357Gln | Shimada, Shimojima et al. 2015(Shimada et al., 2015) |
| 37 | M | 13.0 | *EIF2B5* | c.915G>A | p.Met305Ile | c.1154T>C | p.Ile385Thr | Shimada, Shimojima et al. 2015(Shimada et al., 2015) |
| 38 | F | 0.4 | *EIF2B5* | c.230A>G | p.Asp77Gly | c.407G>A | p.Arg136His | Sharma, Ajij et al. 2015(Sharma et al., 2015) |
| 39 | F | 41.0 | *EIF2B3* | c.260C>T | p.Ala87Val | c.260C>T | p.Ala87Val | Herwerth, Schwaiger et al. 2015(Herwerth et al., 2015) |
| 40 | M | 0.3 | *EIF2B4* | c.1091G>A | p.Arg364Gln | c.1091G>A | p.Arg364Gln | Gungor, Ozkaya et al. 2015(Gungor et al., 2015) |
| 41 | M | 1.5 | *EIF2B3* | c.136G>A | p.Val46Ile | c.136G>A | p.Val46Ile | Turón-Viñas, Pineda et al. 2014(Turón-Viñas et al., 2014) |
| 42 | M | 1.5 | *EIF2B3* | c.136G>A | p.Val46Ile | c.136G>A | p.Val46Ile | Turón-Viñas, Pineda et al. 2014(Turón-Viñas et al., 2014) |
| 43 | F | 1.7 | *EIF2B5* | c.338G>A | p.Arg113His | c.468C>G | p.Ile156Met | Turón-Viñas, Pineda et al. 2014(Turón-Viñas et al., 2014) |
| 44 | F | 1.7 | *EIF2B4* | c.1090C>T | p.Arg364Trp | c.1120C>T | p.Arg374Cys | Turón-Viñas, Pineda et al. 2014(Turón-Viñas et al., 2014) |
| 45 | M | 2.5 | *EIF2B5* | c.314A>G | p.His105Arg | c.338G>A | p.Arg113His | Turón-Viñas, Pineda et al. 2014(Turón-Viñas et al., 2014) |
| 46 | F | 2.3 | *EIF2B5* | c.406C>T | p.Arg136Cys | c.406C>T | p.Arg136Cys | Turón-Viñas, Pineda et al. 2014(Turón-Viñas et al., 2014) |
| 47 | M | 2.7 | *EIF2B5* | c.318A>T | p.Leu106Phe | c.338G>A | p.Arg113His | Turón-Viñas, Pineda et al. 2014(Turón-Viñas et al., 2014) |
| 48 | F | 3.6 | *EIF2B5* | c.896G>A | p.Arg299His | c.943C>T | p.Arg315Cys | Turón-Viñas, Pineda et al. 2014(Turón-Viñas et al., 2014) |
| 49 | F | 2.5 | *EIF2B5* | c.338G>A | p.Arg113His | c.805C>T | p.Arg269X | Turón-Viñas, Pineda et al. 2014(Turón-Viñas et al., 2014) |
| 50 | M | 2.5 | *EIF2B5* | c.318A>T | p.Leu106Phe | c.318A>T | p.Leu106Phe | Turón-Viñas, Pineda et al. 2014(Turón-Viñas et al., 2014) |
| 51 | M | 5.5 | *EIF2B5* | c.338G>A | p.Arg113His | c.338G>A | p.Arg113His | Turón-Viñas, Pineda et al. 2014(Turón-Viñas et al., 2014) |
| 52 | F | 3.5 | *EIF2B5* | c.318A>T | p.Leu106Phe | c.318A>T | p.Leu106Phe | Turón-Viñas, Pineda et al. 2014(Turón-Viñas et al., 2014) |
| 53 | F | 2.7 | *EIF2B5* | c.318A>T | p.Leu106Phe | c.406C>T | p.Arg136Cys | Turón-Viñas, Pineda et al. 2014(Turón-Viñas et al., 2014) |
| 54 | F | 8.0 | *EIF2B5* | c.318A>T | p.Leu106Phe | c.338G>A | p.Arg113His | Turón-Viñas, Pineda et al. 2014(Turón-Viñas et al., 2014) |
| 55 | F | 3.6 | *EIF2B5* | c.338G>A | p.Arg113His | c.338G>A | p.Arg113His | Turón-Viñas, Pineda et al. 2014(Turón-Viñas et al., 2014) |
| 56 | F | 2.5 | *EIF2B5* | c.318A>T | p.Leu106Phe | c.395G>C | p.Gly132Ala | Turón-Viñas, Pineda et al. 2014(Turón-Viñas et al., 2014) |
| 57 | M | 2.4 | *EIF2B3* | c.260C>T | p.Ala87Val | c.260C>T | p.Ala87Val | Robinson, Rossignol et al. 2014(Robinson et al., 2014) |
| 58 | F | 2.1 | *EIF2B3* | c.260C>T | p.Ala87Val | c.260C>T | p.Ala87Val |  |
| 59 | F | 20.0 | *EIF2B3* | c.260C>T | p.Ala87Val | c.272G>A | p.Arg91His |  |
| 60 | M | 3.5 | *EIF2B2* | c.638A>G | p.Glu213Gly | c.638A>G | p.Glu213Gly |  |
| 61 | F | 17.0 | *EIF2B5* | c.338G>A | p.Arg113His | c.338G>A | p.Arg113His |  |
| 62 | F | 1.8 | *EIF2B5* | c.338G>A | p.Arg113His | c.338G>A | p.Arg113His | Klingelhoefer, Misbahuddin et al. 2014(Klingelhoefer et al., 2014) |
| 63 | M | 0.8 | *EIF2B2* | c.254T>A | p.Val85Glu | c.254T>A | p.Val85Glu | Hata, Kinoshita et al. 2014(Hata et al., 2014) |
| 64 | M | 0.2 | *EIF2B2* | c.817A>C | p.Lys273Gln | c.939_948del | p.Asp314Profs*23 | Unal, Ozgen et al. 2013(Unal et al., 2013) |
| 65 | F | 1.5 | *EIF2B2* | c.638A>G | p.Glu213Gly | c.638A>G | p.Glu213Gly | Sambati, Agati et al. 2013(Sambati et al., 2013) |
| 66 | F | 2.5 | *EIF2B5* | c.318A>T | p.Leu106Phe | c.395G>C | p.Gly132Ala | Alías Hernández, Ramos Lizana et al. 2013(Alías Hernández et al., 2013) |
| 67 | F | no onset at 1.0 | *EIF2B5* | c.247delC | p.Leu83X | c.475A>G | p.Ile159Val | van der Lei, Steenweg et al. 2012(van der Lei et al., 2012) |
| 68 | F | 1.5 | *EIF2B5* | c.338G>A | p.Arg113His | c.1208C > T | p.Ala403Val | van der Lei, Steenweg et al. 2012(van der Lei et al., 2012) |
| 69 | F | 1.7 | *EIF2B2* | c.599G>T | p.Gly200Val | c.638A>G | p.Glu213Gly | van der Lei, Steenweg et al. 2012(van der Lei et al., 2012) |
| 70 | F | 3.5 | *EIF2B4* | c.499–1G>C | p.Val167Hisfs*47 | c.626A>G | p.Arg209Gln | van der Lei, Steenweg et al. 2012(van der Lei et al., 2012) |
| 71 | M | 4.4 | *EIF2B5* | c.5C>T | p.Ala2Val | c.631A>G | p.Arg211Gly | van der Lei, Steenweg et al. 2012(van der Lei et al., 2012) |
| 72 | F | 13.2 | *EIF2B5* | c.338G>A | p.Arg113His | c.1946T>C | p.Ile649Thr | van der Lei, Steenweg et al. 2012(van der Lei et al., 2012) |
| 73 | M | 15.8 | *EIF2B2* | c.599G>T | p.Gly200Val | c.880G>T | p.Val294Phe | van der Lei, Steenweg et al. 2012(van der Lei et al., 2012) |
| 74 | M | 7.0 | *EIF2B2* | c.638A>G | p.Glu213Gly | c.638A>G | p.Glu213Gly | Valálik, van der Knaap et al. 2012(Valálik et al., 2012) |
| 75 | F | 61.0 | *EIF2B3* | c. 260C>T | p.Ala87Val | c. 260C>T | p.Ala87Val | Ghezzi, Scarpini et al. 2012(Ghezzi et al., 2012) |
| 76 | M | 0.8 | *EIF2B3* | c.144T>A | p.Phe48Leu | c.144T>A | p.Phe48Leu | Ding, Bley et al. 2012(Ding et al., 2012) |
| 77 | F | 14.0 | *EIF2B4* | c.625C>T | p.Arg209X | c.626G>A | p.Arg209Gln | Ding, Bley et al. 2012(Ding et al., 2012) |
| 78 | F | 19.0 | *EIF2B2* | c.512C>T | p.Ser171Phe | c.599G>T | p.Gly200Val | Ding, Bley et al. 2012(Ding et al., 2012) |
| 79 | F | 16.0 | *EIF2B5* | c.338G>A | p.Arg113His | c.338G>A | p.Arg113His | Damásio, van der Lei et al. 2012(Damásio et al., 2012) |
| 80 | F | 0.8 | *EIF2B2* | c.803G>A | p.Cys268Tyr | c.803G>A | p.Cys268Tyr | Alsalem, Shaheen et al. 2012(Alsalem et al., 2012) |
| 81 | M | 3.5 | *EIF2B5* | c.449T>G | p.Leu150Arg | c.1355A>G | p.His452Arg | Sharma, Arya et al. 2011(Sharma et al., 2011) |
| 82 | M | 9.0 | *EIF2B5* | c.626G>A | p.Arg209Gln | c.1399C>T | p.Arg467Trp | Prange and Weber 2011(Prange and Weber, 2011) |
| 83 | F | 43.0 | *EIF2B2* | c.254T>A | p.Val85Glu | c.254T>A | p.Val85Glu | Matsukawa, Wang et al. 2011(Matsukawa et al., 2011) |
| 84 | M | 50.0 | *EIF2B5* | c.808G>C | p.Asp270His | c.808G>C | p.Asp270His |  |
| 85 | F | 29.0 | *EIF2B3* | c.80T>A | p.Leu27Gln | c.80T>A | p.Leu27Gln |  |
| 86 | F | 6.5 | *EIF2B5* | c.338G> A | p.Arg113His | c.896G>A | p.Arg299His | Imam, Brown et al. 2011)(Imam et al., 2011) |
| 87 | F | 27.0 | *EIF2B5* | c.338G>A | p.Arg113His | c.338G>A | p.Arg113His | Carra-Dalliere, Horzinski et al. 2011(Carra-Dalliere et al., 2011) |
| 88 | F | 17.0 | *EIF2B5* | c.338G>A | p.Arg113His | c.338G>A | p.Arg113His | Carra-Dalliere, Horzinski et al. 2011(Carra-Dalliere et al., 2011) |
| 89 | F | 30.0 | *EIF2B5* | c.338G>A | p.Arg113His | c.338G>A | p.Arg113His | Carra-Dalliere, Horzinski et al. 2011(Carra-Dalliere et al., 2011) |
| 90 | F | 23.0 | *EIF2B5* | c.338G>A | p.Arg113His | c.896G>A | p.Arg299His | Carra-Dalliere, Horzinski et al. 2011(Carra-Dalliere et al., 2011) |
| 91 | M | 62.0 | *EIF2B5* | c.743A>T | p.His248Leu | c.743A>T | p.His248Leu | Carra-Dalliere, Horzinski et al. 2011(Carra-Dalliere et al., 2011) |
| 92 | F | 34.0 | *EIF2B5* | c.584G>A | p.Arg195His | c.1448A>G | p.Tyr483Cys | Carra-Dalliere, Horzinski et al. 2011(Carra-Dalliere et al., 2011) |
| 93 | F | 23.0 | *EIF2B5* | c.338G>A | p.Arg113His | c.338G>A | p.Arg113His | Carra-Dalliere, Horzinski et al. 2011(Carra-Dalliere et al., 2011) |
| 94 | M | 52.0 | *EIF2B5* | c.338G>A | p.Arg113His | c.338G>A | p.Arg113His | Carra-Dalliere, Horzinski et al. 2011(Carra-Dalliere et al., 2011) |
| 95 | F | 46.0 | *EIF2B5* | c.338G>A | p.Arg113His | c.338G>A | p.Arg113His | Carra-Dalliere, Horzinski et al. 2011(Carra-Dalliere et al., 2011) |
| 96 | F | 16.0 | *EIF2B5* | c.338G>A | p.Arg113His | c.338G>A | p.Arg113His | Carra-Dalliere, Horzinski et al. 2011(Carra-Dalliere et al., 2011) |
| 97 | M | 27.0 | *EIF2B5* | c.338G>A | p.Arg113His | c.338G>A | p.Arg113His | Carra-Dalliere, Horzinski et al. 2011(Carra-Dalliere et al., 2011) |
| 98 | F | 21.0 | *EIF2B5* | c.641A>G | p.His214Arg | c.805C>T | p.Arg269X | Carra-Dalliere, Horzinski et al. 2011(Carra-Dalliere et al., 2011) |
| 99 | F | 24.0 | *EIF2B5* | c.338G>A | p.Arg113His | c.664C>T | p.Arg222Trp | Carra-Dalliere, Horzinski et al. 2011(Carra-Dalliere et al., 2011) |
| 100 | F | 35.0 | *EIF2B5* | c.338G>A | p.Arg113His | c.338G>A | p.Arg113His | Carra-Dalliere, Horzinski et al. 2011(Carra-Dalliere et al., 2011) |
| 101 | F | 42.0 | *EIF2B5* | c.338G>A | p.Arg113His | c.338G>A | p.Arg113His | Carra-Dalliere, Horzinski et al. 2011(Carra-Dalliere et al., 2011) |
| 102 | F | 33.0 | *EIF2B5* | c.338G>A | p.Arg113His | c.338G>A | p.Arg113His | Carra-Dalliere, Horzinski et al. 2011(Carra-Dalliere et al., 2011) |
| 103 | F | 41.0 | *EIF2B4* | c.818T>C | p.Met273Thr | c.1346C>T | p.Thr499Ile | Carra-Dalliere, Horzinski et al. 2011(Carra-Dalliere et al., 2011) |
| 104 | F | 16.0 | *EIF2B3* | c.604G>A | p.Ala202Thr | c.1312C>T | p.Arg438X | Carra-Dalliere, Horzinski et al. 2011(Carra-Dalliere et al., 2011) |
| 105 | M | 12.0 | *EIF2B5* | c.338G>A | p.Arg113His | c.338G>A | p.Arg113His | Carra-Dalliere, Horzinski et al. 2011(Carra-Dalliere et al., 2011) |
| 106 | F | 18.0 | *EIF2B5* | c.338G>A | p.Arg113His | c.338G>A | p.Arg113His | Carra-Dalliere, Horzinski et al. 2011(Carra-Dalliere et al., 2011) |
| 107 | M | 37.0 | *EIF2B5* | c.338G>A | p.Arg113His | c.338G>A | p.Arg113His | Carra-Dalliere, Horzinski et al. 2011(Carra-Dalliere et al., 2011) |
| 108 | F | 57.0 | *EIF2B3* | c.41C>T | p.Ser14Phe | c.260C>T | p.Ala87Val | Carra-Dalliere, Horzinski et al. 2011(Carra-Dalliere et al., 2011) |
| 109 | F | 27.0 | *EIF2B5* | c.338G>A | p.Arg113His | c.338G>A | p.Arg113His | Carra-Dalliere, Horzinski et al. 2011(Carra-Dalliere et al., 2011) |
| 110 | M | 0.7 | *EIF2B5* | c.584G>A | p.Arg195His | c.584G>A | p.Arg195His | Harder, Gourgaris et al. 2010(Harder et al., 2010) |
| 111 | F | 0.8 | *EIF2B5* | c.584G>A | p.Arg195His | c.584G>A | p.Arg195His |  |
| 112 | F | 46.0 | *EIF2B5* | c.1340C>T | p.Ser447Leu | c.545C>T | p.Thr182Met | Lee, Koh et al. 2009(Lee et al., 2009) |
| 113 | F | 42.5 | *EIF2B5* | c.338G>A | p.Arg113His | c.338G>A | p.Arg113His | Labauge, Horzinski et al. 2009(Labauge et al., 2009) |
| 114 | F | 33.0 | *EIF2B5* | c.338G>A | p.Arg113His | c.338G>A | p.Arg113His | Labauge, Horzinski et al. 2009(Labauge et al., 2009) |
| 115 | F | 27.0 | *EIF2B5* | c.338G>A | p.Arg113His | c.338G>A | p.Arg113His | Labauge, Horzinski et al. 2009(Labauge et al., 2009) |
| 116 | M | 27.0 | *EIF2B5* | c.338G>A | p.Arg113His | c.338G>A | p.Arg113His | Labauge, Horzinski et al. 2009(Labauge et al., 2009) |
| 117 | F | 21.0 | *EIF2B5* | c.338G>A | p.Arg113His | c.896G>A | p.Arg299His | Labauge, Horzinski et al. 2009(Labauge et al., 2009) |
| 118 | F | 16.0 | *EIF2B2* | c.638A>G | p.Glu213Gly | c.818A>G | p.Lys273His | Labauge, Horzinski et al. 2009(Labauge et al., 2009) |
| 119 | F | 30.0 | *EIF2B5* | c.338G>A | p.Arg113His | c.338G>A | p.Arg113His | Labauge, Horzinski et al. 2009(Labauge et al., 2009) |
| 120 | M | 52.0 | *EIF2B5* | c.338G>A | p.Arg113His | c.338G>A | p.Arg113His | Labauge, Horzinski et al. 2009(Labauge et al., 2009) |
| 121 | F | 46.0 | *EIF2B5* | c.338G>A | p.Arg113His | c.338G>A | p.Arg113His | Labauge, Horzinski et al. 2009(Labauge et al., 2009) |
| 122 | F | 16.0 | *EIF2B5* | c.338G>A | p.Arg113His | c.338G>A | p.Arg113His | Labauge, Horzinski et al. 2009(Labauge et al., 2009) |
| 123 | F | 34.0 | *EIF2B5* | c.584G>A | p.Arg195His | c.1448A>G | p.Tyr483Cys | Labauge, Horzinski et al. 2009(Labauge et al., 2009) |
| 124 | F | 21.0 | *EIF2B5* | c.641A>G | p.His214Arg | c.805C>T | p.Arg269X | Labauge, Horzinski et al. 2009(Labauge et al., 2009) |
| 125 | F | 35.0 | *EIF2B5* | c.338G>A | p.Arg113His | c.338G>A | p.Arg113His | Labauge, Horzinski et al. 2009(Labauge et al., 2009) |
| 126 | F | 17.0 | *EIF2B5* | c.338G>A | p.Arg113His | c.338G>A | p.Arg113His | Labauge, Horzinski et al. 2009(Labauge et al., 2009) |
| 127 | F | 18.0 | *EIF2B5* | c.338G>A | p.Arg113His | c.338G>A | p.Arg113His | Labauge, Horzinski et al. 2009(Labauge et al., 2009) |
| 128 | M | 62.0 | *EIF2B5* | c.743A>T | p.His248Leu | c.743A>T | p.His248Leu | Labauge, Horzinski et al. 2009(Labauge et al., 2009) |
| 129 | NA | 57.0 | *EIF2B3* | c.41C>T | p.Ser14Phe | c.260C>T | p.Ala87Val | Horzinski, Huyghe et al. 2009(Horzinski et al., 2009) |
| 130 | NA | 8.0 | *EIF2B5* | c.338G>A | p.Arg113His | c.338G>A | p.Arg113His | Horzinski, Huyghe et al. 2009(Horzinski et al., 2009) |
| 131 | NA | 5.0 | *EIF2B5* | c.338G>A | p.Arg113His | c.943C>T | p.Arg315Cys | Horzinski, Huyghe et al. 2009(Horzinski et al., 2009) |
| 132 | NA | 18.0 | *EIF2B5* | c.338G>A | p.Arg113His | c.338G>A | p.Arg113His | Horzinski, Huyghe et al. 2009(Horzinski et al., 2009) |
| 133 | NA | 24.0 | *EIF2B5* | c.338G>A | p.Arg113His | NA | p.Arg222X | Horzinski, Huyghe et al. 2009(Horzinski et al., 2009) |
| 134 | NA | 17.0 | *EIF2B3* | c.604G>A | p.Ala202Thr | c.1312C>T | p.Arg438X | Horzinski, Huyghe et al. 2009(Horzinski et al., 2009) |
| 135 | NA | 7.0 | *EIF2B5* | NA | p.Pro87Leu | c.338G>A | p.Arg113His | Horzinski, Huyghe et al. 2009(Horzinski et al., 2009) |
| 136 | NA | 25.0 | *EIF2B5* | c.1448A>G | p.Tyr483Cys | c.584G>A | p.Arg195His | Horzinski, Huyghe et al. 2009(Horzinski et al., 2009) |
| 137 | NA | 28.0 | *EIF2B5* | g.IVS8+59A/G/? | p.? | g.IVS8+59A/G/? | p.? | Horzinski, Huyghe et al. 2009(Horzinski et al., 2009) |
| 138 | NA | 3.5 | *EIF2B5* | c.338G>A | p.Arg113His | c.[+2081delG] | p.? | Horzinski, Huyghe et al. 2009(Horzinski et al., 2009) |
| 139 | NA | 2.0 | *EIF2B5* | c.1336C>T | p.Pro427Leu | c.1336C>T | p.Pro427Leu | Horzinski, Huyghe et al. 2009(Horzinski et al., 2009) |
| 140 | NA | 2.8 | *EIF2B5* | c.318A>T | p.Leu106Phe | c.338G>A | p.Arg113His | Horzinski, Huyghe et al. 2009(Horzinski et al., 2009) |
| 141 | NA | 22.0 | *EIF2B5* | c.641A>G | p.His214Arg | c.805C>T | p.Arg269X | Horzinski, Huyghe et al. 2009(Horzinski et al., 2009) |
| 142 | F | 55.0 | *EIF2B5* | c.338G>A | p.Arg113His | c.338G>A | p.Arg113His | Gascon-Bayarri, Campdelacreu et al. 2009(Gascon-Bayarri et al., 2009) |
| 143 | F | 4.0 | *EIF2B4* | c.1120C>T | p.Arg374Cys | c.1120C>T | p.Arg374Cys | Wong, Luk et al. 2008(Wong et al., 2008) |
| 144 | F | 2.0 | *EIF2B5* | c.338G>A | p.Arg113His | c.468G>C | p.Ile156Met | Pineda, A et al. 2008(Pineda et al., 2008) |
| 145 | F | 18.0 | *EIF2B5* | c.338G>A | p.Arg113His | c.338G>A | p.Arg113His | Mathis, Scheper et al. 2008(Mathis et al., 2008) |
| 146 | M | 8.0 | *EIF2B5* | c.338G>A | p.Arg113His | c.338G>A | p.Arg113His | Jansen, Andermann et al. 2008(Jansen et al., 2008) |
| 147 | F | 3.5 | *EIF2B5* | c.338G>A | p.Arg113His | c.766-1G>A | p.256_281del | Horzinski, Gonthier et al. 2008(Horzinski et al., 2008) |
| 148 | F | 3.5 | *EIF2B5* | c.338G>A | p.Arg113His | c.766-1G>A | p.256_281del | Horzinski, Gonthier et al. 2008(Horzinski et al., 2008) |
| 149 | M | 8.8 | *EIF2B5* | c.338G>A | p.Arg113His | NA | p.Met608lle | Fontenelle, Scheper et al. 2008(Fontenelle et al., 2008) |
| 150 | M | 2.3 | *EIF2B3* | NA | p.Val30Gly | NA | p.Ala87Val | Dreha-Kulaczewski, Dechent et al. 2008(Dreha-Kulaczewski et al., 2008) |
| 151 | M | 3.6 | *EIF2B3* | NA | p.Arg226Gln | NA | p.Arg226Gln | Dreha-Kulaczewski, Dechent et al. 2008(Dreha-Kulaczewski et al., 2008) |
| 152 | F | 44.0 | *EIF2B5* | c.338G>A | p.Arg113His | c.338G>A | p.Arg113His | Damon-Perriere, Menegon et al. 2008(Damon-Perriere et al., 2008) |
| 153 | F | 20.0 | *EIF2B5* | c.338G>A | p.Arg113His | c.338G>A | p.Arg113His | Damon-Perriere, Menegon et al. 2008(Damon-Perriere et al., 2008) |
| 154 | M | 14.0 | *EIF2B5* | c.338G>A | p.Arg113His | c.338G>A | p.Arg113His | Riecker, Nägele et al. 2007(Riecker et al., 2007) |
| 155 | F | 42.0 | *EIF2B5* | c.1459G>A | p.Glu487Lys | c.1459G>A | p. Glu487Lys | Matsui, Mizutani et al. 2007(Matsui et al., 2007) |
| 156 | M | 8.0 | *EIF2B5* | c.338G>A | p.Arg113His | c.338G>A | p.Arg113His | Lucas, Suarez et al. 2007(Lucas et al., 2007) |
| 157 | M | 0.9 | *EIF2B5* | c.584G>A | p.Arg195His | c.584G>A | p.Arg195His | Huntsman, Seshia et al. 2007(Huntsman et al., 2007) |
| 158 | M | 27.0 | *EIF2B5* | c.338G>A | p.Arg113His | c.338G>A | p.Arg113His | Denier, Orgibet et al. 2007(Denier et al., 2007) |
| 159 | M | 8.0 | *EIF2B5* | c.338G>A | p.Arg113His | c.338G>A | p.Arg113His | Ramaswamy, Chan et al. 2006(Ramaswamy et al., 2006) |
| 160 | F | 4.0 | *EIF2B2* | c.638A>G | p.Glu213Gly | c.638A>G | p.Glu213Gly | Mierzewska, van der Knaap et al. 2006(Mierzewska et al., 2006) |
| 161 | F | 2.0 | *EIF2B2* | c.638A>G | p.Glu213Gly | c.638A>G | p.Glu213Gly | Mierzewska, van der Knaap et al. 2006(Mierzewska et al., 2006) |
| 162 | F | 6.0 | *EIF2B2* | c.638A>G | p.Glu213Gly | c.638A>G | p.Glu213Gly | Mierzewska, van der Knaap et al. 2006(Mierzewska et al., 2006) |
| 163 | M | 9.0 | *EIF2B5* | c.338G>A | p.Arg113His | c.338G>A | p.Arg113His | Mascalchi, De Grandis et al. 2006(Mascalchi et al., 2006) |
| 164 | F | 3.0 | *EIF2B5* | c.338G>A | p.Arg113His | c.1810C>T | p.Pro604Ser | Kaczorowska, Kuczynski et al. 2006(Kaczorowska et al., 2006) |
| 165 | M | 3.0 | *EIF2B5* | c.203T>C | p.Leu68Ser | c.806G>A | p.Arg269Gln | Federico, Scali et al. 2006(Federico et al., 2006) |
| 166 | F | 2.5 | *EIF2B5* | c.1813delC | p.Leu605fs*18 | c.338G>A | p.Arg113His | Wilson, Pronk et al. 2005(Wilson et al., 2005) |
| 167 | M | 2.0 | *EIF2B5* | c.271A>G | p.Thr91Ala | c.271A>G | p.Thr91Ala | Vermeulen, Seidl et al. 2005(Vermeulen et al., 2005) |
| 168 | M | 3.7 | *EIF2B5* | c.331T>C | p.Trp111Arg | c.1360C>T | p.Phe454Ser | Vermeulen, Seidl et al. 2005(Vermeulen et al., 2005) |
| 169 | F | 10.0 | *EIF2B1* | c.547G>T | p.Val183Phe | c.547G>T | p.Val183Phe | Ohlenbusch, Henneke et al. 2005(Ohlenbusch et al., 2005) |
| 170 | F | 17.0 | *EIF2B1* | c.547G>T | p.Val183Phe | c.547G>T | p.Val183Phe | Ohlenbusch, Henneke et al. 2005(Ohlenbusch et al., 2005) |
| 171 | F | 2.0 | *EIF2B2* | c.607_612delinsTG | p.Met203fs*1 | c.638A>G | p.Glu213Gly | Ohlenbusch, Henneke et al. 2005(Ohlenbusch et al., 2005) |
| 172 | F | 22.0 | *EIF2B2* | c.512C>T | p.Ser171Phe | c.599G>T | p.Gly200Val | Ohlenbusch, Henneke et al. 2005(Ohlenbusch et al., 2005) |
| 173 | F | 4.0 | *EIF2B3* | c.674G>A | p.Arg225Gln | c.674G>A | p.Arg225Gln | Ohlenbusch, Henneke et al. 2005(Ohlenbusch et al., 2005) |
| 174 | F | 14.0 | *EIF2B4* | c.625C>T | p.Arg209X | c.626G>A | p.Arg209Gln | Ohlenbusch, Henneke et al. 2005(Ohlenbusch et al., 2005) |
| 175 | F | 2.0 | *EIF2B5* | c.338G>A | p.Arg113His | c.929G>T | p.Cys310Phe | Ohlenbusch, Henneke et al. 2005(Ohlenbusch et al., 2005) |
| 176 | F | 1.0 | *EIF2B5* | c.203T>C | p.Leu68Ser | c.453_454del | p.Tyr152fs*12 | Ohlenbusch, Henneke et al. 2005(Ohlenbusch et al., 2005) |
| 177 | F | 17.0 | *EIF2B2* | c.638A>G | p.Glu213Gly | c.638A>G | p.Glu213Gly | Jurkiewicz, Mierzewska et al. 2005(Jurkiewicz et al., 2005) |
| 178 | F | 9.0 | *EIF2B2* | c.638A>G | p.Glu213Gly | c.638A>G | p.Glu213Gly | Jurkiewicz, Mierzewska et al. 2005(Jurkiewicz et al., 2005) |
| 179 | F | 7.0 | *EIF2B2* | c.638A>G | p.Glu213Gly | c.638A>G | p.Glu213Gly | Jurkiewicz, Mierzewska et al. 2005(Jurkiewicz et al., 2005) |
| 180 | F | 25.0 | *EIF2B5* | c.338G>A | p.Arg113His | c.338G>A | p.Arg113His | van der Knaap, Leegwater et al. 2004(van der Knaap et al., 2004) |
| 181 | M | 9.0 | *EIF2B5* | c.338G>A | p.Arg113His | c.338G>A | p.Arg113His | van der Knaap, Leegwater et al. 2004(van der Knaap et al., 2004) |
| 182 | F | not yet at 30.0 | *EIF2B5* | c.338G>A | p.Arg113His | c.338G>A | p.Arg113His | van der Knaap, Leegwater et al. 2004(van der Knaap et al., 2004) |
| 183 | F | 27.0 | *EIF2B5* | c.338G>A | p.Arg113His | c.338G>A | p.Arg113His | van der Knaap, Leegwater et al. 2004(van der Knaap et al., 2004) |
| 184 | F | not yet at 20.0 | *EIF2B5* | c.338G>A | p.Arg113His | c.338G>A | p.Arg113His | van der Knaap, Leegwater et al. 2004(van der Knaap et al., 2004) |
| 185 | M | 3.0 | *EIF2B5* | c.338G>A | p.Arg113His | c.338G>A | p.Arg113His | van der Knaap, Leegwater et al. 2004(van der Knaap et al., 2004) |
| 186 | F | 40.0 | *EIF2B5* | c.573C>T | p.Thr182Met | c.573C>T | p.Thr182Met | Ohtake, Shimohata et al. 2004(Ohtake et al., 2004) |
| 187 | M | 0.8 | *EIF2B5* | c.967C>T | p.Pro323Ser | c.1280C>T | p.Phe427Leu | Fogli, Schiffmann et al. 2004(Fogli et al., 2004) |
| 188 | F | 0.8 | *EIF2B5* | c.166T>G | p.Phe56Val | c.944G>A | p.Arg315His | Fogli, Schiffmann et al. 2004(Fogli et al., 2004) |
| 189 | M | 1.2 | *EIF2B5* | c.166T>G | p.Phe56Val | c.944G>A | p.Arg315His | Fogli, Schiffmann et al. 2004(Fogli et al., 2004) |
| 190 | M | 1.0 | *EIF2B5* | c.338G>A | p.Arg113His | c.1274T>G | p.Leu425Arg | Fogli, Schiffmann et al. 2004(Fogli et al., 2004) |
| 191 | F | 1.5 | *EIF2B5* | c.338G>A | p.Arg113His | c.1160T>G | p.Asp387Gly | Fogli, Schiffmann et al. 2004(Fogli et al., 2004) |
| 192 | F | 1.0 | *EIF2B5* | c.1028A>G | p.Tyr343Cys | c.1153A>G | p.Ile385Val | Fogli, Schiffmann et al. 2004(Fogli et al., 2004) |
| 193 | F | 3.0 | *EIF2B5* | c.1028A>G | p.Tyr343Cys | c.1153A>G | p.Ile385Val | Fogli, Schiffmann et al. 2004(Fogli et al., 2004) |
| 194 | M | 2.0 | *EIF2B5* | c.338G>A | p.Arg113His | c.1160T>G | p.Asp387Gly | Fogli, Schiffmann et al. 2004(Fogli et al., 2004) |
| 195 | F | 2.5 | *EIF2B5* | c.338G>A | p.Arg113His | c.1948G>A | p.Glu650Leu | Fogli, Schiffmann et al. 2004(Fogli et al., 2004) |
| 196 | M | 2.5 | *EIF2B5* | c.338G>A | p.Arg113His | c.1948G>A | p.Glu650Leu | Fogli, Schiffmann et al. 2004(Fogli et al., 2004) |
| 197 | M | 2.0 | *EIF2B5* | c.406C>T | p.Arg136Cys | c.1015C>T | p.Arg339Trp | Fogli, Schiffmann et al. 2004(Fogli et al., 2004) |
| 198 | M | 2.0 | *EIF2B5* | c.271A>G | p.Thr91Ala | c.1015C>T | p.Arg339Trp | Fogli, Schiffmann et al. 2004(Fogli et al., 2004) |
| 199 | M | 1.5 | *EIF2B5* | c.271A>G | p.Thr91Ala | c.1015C>T | p.Arg339Trp | Fogli, Schiffmann et al. 2004(Fogli et al., 2004) |
| 200 | F | 2.0 | *EIF2B5* | c.271A>G | p.Thr91Ala | c.1015C>T | p.Arg339Trp | Fogli, Schiffmann et al. 2004(Fogli et al., 2004) |
| 201 | F | 3.0 | *EIF2B5* | c.338G>A | p.Arg113His | c.967C>T | p.Pro323Ser | Fogli, Schiffmann et al. 2004(Fogli et al., 2004) |
| 202 | F | 3.0 | *EIF2B5* | c.338G>A | p.Arg113His | c.1884G>A | p.Trp628X | Fogli, Schiffmann et al. 2004(Fogli et al., 2004) |
| 203 | M | 3.5 | *EIF2B5* | c.338G>A | p.Arg113His | c.806G>T | p.Arg269Leu | Fogli, Schiffmann et al. 2004(Fogli et al., 2004) |
| 204 | M | 3.0 | *EIF2B5* | c.943C>T | p.Arg315Cys | c.271A>G | p.Thr91Ala | Fogli, Schiffmann et al. 2004(Fogli et al., 2004) |
| 205 | 女 | 2.0 | *EIF2B5* | c.592G>A | p.Glu198Lys | c.1996del21 | p.664del7 | Fogli, Schiffmann et al. 2004(Fogli et al., 2004) |
| 206 | M | 3.0 | *EIF2B5* | c.338G>A | p.Arg113His | c.1015C>T | p.Arg339Trp | Fogli, Schiffmann et al. 2004(Fogli et al., 2004) |
| 207 | M | 2.5 | *EIF2B5* | c.338G>A | p.Arg113His | c.1016C>T | c.Arg339Pro | Fogli, Schiffmann et al. 2004(Fogli et al., 2004) |
| 208 | M | 3.5 | *EIF2B5* | c.47C>A | p.Ala16Asp | c.338G>A | p.Arg113His | Fogli, Schiffmann et al. 2004(Fogli et al., 2004) |
| 209 | F | 3.0 | *EIF2B5* | c.338G>A | p.Arg113His | c.1264C>T | p.Arg422X | Fogli, Schiffmann et al. 2004(Fogli et al., 2004) |
| 210 | M | 2.0 | *EIF2B5* | c.338G>A | p.Arg113His | c.1444Gins17 | p.Gly481fs493X | Fogli, Schiffmann et al. 2004(Fogli et al., 2004) |
| 211 | F | 2.0 | *EIF2B5* | c.241G>A | p.Glu81Lys | c.338G>A | p.Arg113His | Fogli, Schiffmann et al. 2004(Fogli et al., 2004) |
| 212 | F | 3.0 | *EIF2B5* | c.241G>A | p.Glu81Lys | c.338G>A | p.Arg113His | Fogli, Schiffmann et al. 2004(Fogli et al., 2004) |
| 213 | F | 2.0 | *EIF2B5* | c.241G>A | p.Glu81Lys | c.338G>A | p.Arg113His | Fogli, Schiffmann et al. 2004(Fogli et al., 2004) |
| 214 | F | 3.0 | *EIF2B5* | c.241G>A | p.Glu81Lys | c.338G>A | p.Arg113His | Fogli, Schiffmann et al. 2004(Fogli et al., 2004) |
| 215 | F | 3.5 | *EIF2B5* | c.338G>A | p.Arg113His | c.584G>A | p.Arg195His | Fogli, Schiffmann et al. 2004(Fogli et al., 2004) |
| 216 | F | 4.5 | *EIF2B5* | c.338G>A | p.Arg113His | c.584G>A | p.Arg195His | Fogli, Schiffmann et al. 2004(Fogli et al., 2004) |
| 217 | M | 2.5 | *EIF2B5* | c.241G>A | p.Glu81Lys | c.338G>A | p.Arg113His | Fogli, Schiffmann et al. 2004(Fogli et al., 2004) |
| 218 | M | 5.0 | *EIF2B5* | c.338G>A | p.Arg113His | c.338G>A | p.Arg113His | Fogli, Schiffmann et al. 2004(Fogli et al., 2004) |
| 219 | F | 3.5 | *EIF2B5* | c.338G>A | p.Arg113His | c.338G>A | p.Arg113His | Fogli, Schiffmann et al. 2004(Fogli et al., 2004) |
| 220 | M | 4.0 | *EIF2B5* | c.338G>A | p.Arg113His | c.338G>A | p.Arg113His | Fogli, Schiffmann et al. 2004(Fogli et al., 2004) |
| 221 | F | 3.0 | *EIF2B5* | c.338G>A | p.Arg113His | c.338G>A | p.Arg113His | Fogli, Schiffmann et al. 2004(Fogli et al., 2004) |
| 222 | F | 4.0 | *EIF2B5* | c.338G>A | p.Arg113His | c.338G>A | p.Arg113His | Fogli, Schiffmann et al. 2004(Fogli et al., 2004) |
| 223 | M | 4.5 | *EIF2B5* | c.338G>A | p.Arg113His | c.338G>A | p.Arg113His | Fogli, Schiffmann et al. 2004(Fogli et al., 2004) |
| 224 | F | 10.0 | *EIF2B5* | c.338G>A | p.Arg113His | c.338G>A | p.Arg113His | Fogli, Schiffmann et al. 2004(Fogli et al., 2004) |
| 225 | F | 6.0 | *EIF2B5* | c.338G>A | p.Arg113His | c.338G>A | p.Arg113His | Fogli, Schiffmann et al. 2004(Fogli et al., 2004) |
| 226 | M | 10.0 | *EIF2B5* | c.338G>A | p.Arg113His | c.338G>A | p.Arg113His | Fogli, Schiffmann et al. 2004(Fogli et al., 2004) |
| 227 | M | 7.0 | *EIF2B5* | c.338G>A | p.Arg113His | c.338G>A | p.Arg113His | Fogli, Schiffmann et al. 2004(Fogli et al., 2004) |
| 228 | M | 9.0 | *EIF2B5* | c.161G>C | p.Arg54Pro | c.337C>T | p.Arg113Cys | Fogli, Schiffmann et al. 2004(Fogli et al., 2004) |
| 229 | M | 8.0 | *EIF2B5* | c.218T>G | p.Val73Glu | c.338G>A | p.Arg113His | Fogli, Schiffmann et al. 2004(Fogli et al., 2004) |
| 230 | M | 7.0 | *EIF2B5* | c.218T>G | p.Val73Glu | c.338G>A | p.Arg113His | Fogli, Schiffmann et al. 2004(Fogli et al., 2004) |
| 231 | F | 16.0 | *EIF2B5* | c.338G>A | p.Arg113His | c.583C>T | p.Arg195Cys | Fogli, Schiffmann et al. 2004(Fogli et al., 2004) |
| 232 | M | 1.5 | *EIF2B4* | c.1069C>T | p.Arg357Trp | c.1069C>T | p.Arg357Trp | Fogli, Schiffmann et al. 2004(Fogli et al., 2004) |
| 233 | M | 2.5 | *EIF2B4* | c.626G>A | p.Arg209Gln | c.626G>A | p.Arg209Gln | Fogli, Schiffmann et al. 2004(Fogli et al., 2004) |
| 234 | M | 1.5 | *EIF2B4* | c.626G>A | p.Arg209Gln | c.626G>A | p.Arg209Gln | Fogli, Schiffmann et al. 2004(Fogli et al., 2004) |
| 235 | M | 6.0 | *EIF2B4* | c.728C>T | p.Pro243Leu | c.728C>T | p.Pro243Leu | Fogli, Schiffmann et al. 2004(Fogli et al., 2004) |
| 236 | F | 3.0 | *EIF2B4* | c.728C>T | p.Pro243Leu | c.728C>T | p.Pro243Leu | Fogli, Schiffmann et al. 2004(Fogli et al., 2004) |
| 237 | F | 4.5 | *EIF2B4* | c.728C>T | p.Pro243Leu | c.728C>T | p.Pro243Leu | Fogli, Schiffmann et al. 2004(Fogli et al., 2004) |
| 238 | F | 3.0 | *EIF2B4* | c.728C>T | p.Pro243Leu | c.728C>T | p.Pro243Leu | Fogli, Schiffmann et al. 2004(Fogli et al., 2004) |
| 239 | M | 4.0 | *EIF2B4* | c.1120C>T | p.Arg374Cys | c.1465T>C | p.Tyr489His | Fogli, Schiffmann et al. 2004(Fogli et al., 2004) |
| 240 | F | 8.0 | *EIF2B4* | c.1120C>T | p.Arg374Cys | c.1120C>T | p.Arg374Cys | Fogli, Schiffmann et al. 2004(Fogli et al., 2004) |
| 241 | M | 4.0 | *EIF2B3* | c.1023T>G | p.His341Gln | c.1023T>G | p.His341Gln | Fogli, Schiffmann et al. 2004(Fogli et al., 2004) |
| 242 | M | 5.0 | *EIF2B3* | c.407A>C | p.Gln136Pro | c.407A>C | p.Gln136Pro | Fogli, Schiffmann et al. 2004(Fogli et al., 2004) |
| 243 | M | 1.5 | *EIF2B2* | c.638A>G | p.Glu213Gly | c.910G>C | p.Glu304X | Fogli, Schiffmann et al. 2004(Fogli et al., 2004) |
| 244 | M | 1.0 | *EIF2B2* | c.638A>G | p.Glu213Gly | c.910G>C | p.Glu304X | Fogli, Schiffmann et al. 2004(Fogli et al., 2004) |
| 245 | M | 5.0 | *EIF2B2* | c.638A>G | p.Glu213Gly | c.638A>G | p.Glu213Gly | Fogli, Schiffmann et al. 2004(Fogli et al., 2004) |
| 246 | F | 5.0 | *EIF2B2* | c.638A>G | p.Glu213Gly | c.638A>G | p.Glu213Gly | Fogli, Schiffmann et al. 2004(Fogli et al., 2004) |
| 247 | M | 5.0 | *EIF2B2* | c.638A>G | p.Glu213Gly | c.638A>G | p.Glu213Gly | Fogli, Schiffmann et al. 2004(Fogli et al., 2004) |
| 248 | M | 4.0 | *EIF2B2* | c.638A>G | p.Glu213Gly | c.638A>G | p.Glu213Gly | Fogli, Schiffmann et al. 2004(Fogli et al., 2004) |
| 249 | M | 2.0 | *EIF2B2* | c.638A>G | p.Glu213Gly | c.638A>G | p.Glu213Gly | Fogli, Schiffmann et al. 2004(Fogli et al., 2004) |
| 250 | M | 7.0 | *EIF2B2* | c.638A>G | p.Glu213Gly | c.638A>G | p.Glu213Gly | Fogli, Schiffmann et al. 2004(Fogli et al., 2004) |
| 251 | M | 7.0 | *EIF2B2* | c.638A>G | p.Glu213Gly | c.638A>G | p.Glu213Gly | Fogli, Schiffmann et al. 2004(Fogli et al., 2004) |
| 252 | M | 14.0 | *EIF2B2* | c.638A>G | p.Glu213Gly | c.638A>G | p.Glu213Gly | Fogli, Schiffmann et al. 2004(Fogli et al., 2004) |
| 253 | M | 7.0 | *EIF2B2* | c.638A>G | p.Glu213Gly | c.818A>G | p.Lys273Arg | Fogli, Schiffmann et al. 2004(Fogli et al., 2004) |
| 254 | F | 17.0 | *EIF2B2* | c.638A>G | p.Glu213Gly | c.818A>G | p.Lys273Arg | Fogli, Schiffmann et al. 2004(Fogli et al., 2004) |
| 255 | M | 4.0 | *EIF2B2* | c.599G>C | p.Gly200Ala | c.638A>G | p.Glu213Gly | Fogli, Schiffmann et al. 2004(Fogli et al., 2004) |
| 256 | F | 0.3 | *EIF2B2* | c.599G>T | p.Gly200Val | c.871C>T | p.Pro291Ser | van der Knaap, van Berkel et al. 2003(van der Knaap et al., 2003) |
| 257 | F | 0.3 | *EIF2B2* | c.599G>T | p.Gly200Val | c.871C>T | p.Pro291Ser | van der Knaap, van Berkel et al. 2003(van der Knaap et al., 2003) |
| 258 | F | 0.3 | *EIF2B2* | c.599G>T | p.Gly200Val | c.871C>T | p.Pro291Ser | van der Knaap, van Berkel et al. 2003(van der Knaap et al., 2003) |
| 259 | M | 0 | *EIF2B4* | c.1447C>T | p.Arg483Trp | c.1447C>T | p.Arg483Trp | van der Knaap, van Berkel et al. 2003(van der Knaap et al., 2003) |
| 260 | F | 0 | *EIF2B4* | c.1447C>T | p.Arg483Trp | c.1447C>T | p.Arg483Trp | van der Knaap, van Berkel et al. 2003(van der Knaap et al., 2003) |
| 261 | F | 0 | *EIF2B4* | c.1172C>A | p.Ala391Asp | c.1172C>A | p.Ala391Asp | van der Knaap, van Berkel et al. 2003(van der Knaap et al., 2003) |
| 262 | M | 5.0 | *EIF2B5* | c.1289T>C | p.Val430Ala | c.1340C>T | p.Ser447Leu | van der Knaap, van Berkel et al. 2003(van der Knaap et al., 2003) |
| 263 | M | 6.0 | *EIF2B5* | c.1484A>G | p.Tyr495Cys | c.1484A>G | p.Tyr495Cys | van der Knaap, van Berkel et al. 2003(van der Knaap et al., 2003) |
| 264 | F | 0.6 | *EIF2B5* | c.584G>A | p.Arg195His | c.584G>A | p.Arg195His | Fogli, Wong et al. 2002(Fogli et al., 2002b) |
| 265 | M | 0.4 | *EIF2B5* | c.584G>A | p.Arg195His | c.584G>A | p.Arg195His | Fogli, Wong et al. 2002(Fogli et al., 2002b) |
| 266 | F | 0.3 | *EIF2B5* | c.584G>A | p.Arg195His | c.584G>A | p.Arg195His | Fogli, Wong et al. 2002(Fogli et al., 2002b) |
| 267 | F | 0.9 | *EIF2B5* | c.925G>C | p.Val309Leu | c. 925G>C | p.Val309Leu | Fogli, Dionisi-Vici et al. 2002(Fogli et al., 2002a) |
| 268 | F | 0.8 | *EIF2B5* | c.925G>C | p.Val309Leu | c. 925G>C | p.Val309Leu | Fogli, Dionisi-Vici et al. 2002(Fogli et al., 2002a) |
| 269 | F | 0.7 | *EIF2B5* | c.806G>A | p.Arg269Gln | c. 1340C>T | p.Ser447Leu | 丁乐, 郭虎 et al. 2016(丁乐 et al., 2016) |
| 270 | M | 2.0 | *EIF2B2* | c.254T>A | p.Val85Glu | c.911_913del | p.305del | 丁乐, 郭虎 et al. 2016(丁乐 et al., 2016) |
| 271 | M | 0.1 | *EIF2B5* | c.1016G>A | p.Arg339Gln | c.1809delC | p.Phe603fs*2 | 张晓莉, 王丽君 et al. 2017(张晓莉 et al., 2017) |
| 272 | M | 1.1 | *EIF2B5* | c.1015C>T | p.Arg339Trp | c.1015C>T | p.Arg339Trp | 马慧 and 林剑军 2019(马慧 and 林剑军, 2019) |
| 273 | M | 0.8 | *EIF2B5* | c.232T>C | p.Tyr78His | c.407G>A | p.Arg136His | 莫庭庭, 李霞 et al. 2020(莫庭庭 et al., 2020) |
| 274 | F | 1.3 | *EIF2B5* | c.407G>A | p.Arg136His | c.944G>A | p.Arg315His | 徐慧, 刘开运 et al. 2019(徐慧 et al., 2019) |
| 275 | F | 8m | *EIF2B5* | c.806G>A | p.Arg269Gln | c.1340C>T | p.Ser447Leu | 徐化凤, 管红梅 et al. 2019(徐化凤 et al., 2019) |
| 276 | F | 5.2 | *EIF2B2* | c.233T>A | p.Val78Glu | c.535_536del | p.Lys179Argfs*23 | 徐化凤, 管红梅 et al. 2019(徐化凤 et al., 2019) |
| 277 | M | 2.2 | *EIF2B2* | c.254T>A | p.Val85Glu | c.911_913del | p.305del | 徐化凤, 管红梅 et al. 2019(徐化凤 et al., 2019) |
| 278 | F | 17.0 | *EIF2B5* | c.915G>A | p.Met305Ile | c.1484A>G | p.Tyr495Cys | 李霓, 林楠 et al. 2018(李霓 et al., 2018) |
| 279 | M | 2.0 | *EIF2B3* | c.674G >A | p.Arg225Gln | c.674G >A | p.Arg225Gln | 刘宇, 王芳 et al. 2016(刘宇 et al., 2016) |
| 280 | M | 2.6 | *EIF2B5* | c.943C >T | p.Arg315Cys | c.943C >T | p.Arg315Cys | 刘宇, 王芳 et al. 2016(刘宇 et al., 2016) |
| 281 | M | 3.4 | *EIF2B5* | c.536C >T | p.Ser179Phe | c.536C >T | p.Ser179Phe | 刘宇, 王芳 et al. 2016(刘宇 et al., 2016) |
| 282 | F | 3.0 | *EIF2B3* | c.140G>A | p.Gly47Glu | c.1037T>C | p.Ile346Thr |  |
| 283 | M | 1.3 | *EIF2B5* | c.943C>T | p.Arg315Cys | c.943C>T | p.Arg315Cys |  |
| 284 | M | 1.5 | *EIF2B5* | c.1340C>T | p.Ser447Leu | c.1126A>G | p.Asn376Asp |  |
| 285 | F | 2.0 | *EIF2B5* | c.805C>T | p.Arg269X | c.1004G>C | p.Cys335Ser |  |
| 286 | F | 2.3 | *EIF2B5* | c.805C>T | p.Arg269X | c.1004G>C | p.Cys335Ser |  |
| 287 | M | 3.7 | *EIF2B3* | c.674G>A | p.Arg225Gln | c.674G>A | p.Arg225Gln |  |
| 288 | M | 6.4 | *EIF2B5* | c.185A>T | p.Asp62Val | c.1016G>C | p.Arg339Pro |  |
| 289 | M | 4.3 | *EIF2B5* | c.1827_1838del | p.610_613del4 | c.1157G>A | P.Gly386Val |  |
| 290 | M | 5.0 | *EIF2B3* | c.1037T>C | p.Ile346Thr | c.1037T>C | p.Ile346Thr |  |
| 291 | M | 3.5 | *EIF2B2* | c.254T>A | p.Val85Glu | c.817A>C | p.Lys273Gln |  |
| 292 | 1 | 2.4 | *EIF2B5* | c.337C>A | p.Arg113Cys | c.806G>A | p.Arg269Gln |  |
| 293 | M | 4.3 | *EIF2B3* | c.1037T>C | p.Ile346Thr | c.1037T>C | p.Ile346Thr |  |
| 294 | F | 1.9 | *EIF2B2* | c.254T>A | p.Val85Glu | c.922G>A | p.Val308Met |  |
| 295 | F | 4.5 | *EIF2B5* | c.806G>A | p.Arg269Gln | c.915G>A | P.Met305Ile |  |
| 296 | F | 1.7 | *EIF2B4* | c.1180C>T | p.Leu394phe | c.1180C>T | p.Leu394phe |  |
| 297 | M | 2.5 | *EIF2B3* | c.674G>A | p.Arg225Gln | c.674G>A | p.Arg225Gln |  |
| 298 | M | 1.6 | *EIF2B3* | c.935G>A | p.Arg312Gln | c.1037T>C | p.Ile346Thr |  |
| 299 | M | 5.0 | *EIF2B4* | c.407A>G | p.Gln136Arg | c.407A>G | p.Gln136Arg |  |
| 300 | M | 0.5 | *EIF2B4* | c.691G>A | p.Gly231Ser | c.1459C>T | p.Arg487Trp |  |
| 301 | M | 2.3 | *EIF2B3* | c.1037T>C | p.Ile346Thr | c.1106-1113del | p.Ser369Cysfs*404 |  |
| 302 | M | 5.0 | *EIF2B4* | c.1382A>G | p.Tyr461Cys | c.1565C>T | p.Thr522Met |  |
| 303 | M | 5.3 | *EIF2B4* | c.1180C>T | p.Leu394Phe | c.1180C>T | p.Leu394Phe |  |
| 304 | F | 1.6 | *EIF2B5* | c.806G>A | p.Arg269Gln | c.806G>A | p.Arg269Gln |  |
| 305 | M | 7.5 | *EIF2B2* | c.818A>G | p.Lys273Arg | c.922G>A | p. Val308Met |  |
| 306 | M | 2.5 | *EIF2B5* | c.943C>T | p.Arg315Cys | c.943C>T | p.Arg315Cys |  |
| 307 | F | 2.2 | *EIF2B1* | c.328A>G | p.Lys110Glu | c.328A>G | p.Lys110Glu |  |
| 308 | F | 1.8 | *EIF2B5* | c.185A>T | p.Asp62Val | c.1518delA | p.Glu506_fs*52 |  |
| 309 | F | 1.4 | *EIF2B3* | c.32G>T | p.Gly11Val | c.32G>T | p.Gly11Val |  |
| 310 | M | 0.6 | *EIF2B5* | c.1340C>T | p.Ser447Leu | c.1340C>T | p.Ser447Leu |  |
| 311 | M | 3.0 | *EIF2B2* | c.254T>A | p.Val85Glu | c.995C>T | p.Ala332Val |  |
| 312 | M | 9.6 | *EIF2B4* | c.1306T>A | p.Ser416Thr | c.1397G>A | p.Arg446His |  |
| 313 | M | 2.1 | *EIF2B5* | c.947G>A | p.Arg316Gln | c.1352T>C | p.Leu451Ser |  |
| 314 | M | 3.6 | *EIF2B3* | c.965C>G | p.Ala322Gly | c.1037T>C | p.Ile346Thr |  |
| 315 | F | 2.8 | *EIF2B5* | c.583C>T | p.Arg195Cys | c.915G>A | p.Met305Ile |  |
| 316 | F | 2.9 | *EIF2B2* | c.254T>A | p.Val85Glu | c.817A>C | p.Lys273Gln |  |
| 317 | M | 3.8 | *EIF2B3* | c.965C>G | p.Ala322Gly | c.1037T>C | p.Ile346Thr |  |
| 318 | F | 0.7 | *EIF2B4* | c.815T>C | p.Met272Thr | c.820A>T | p.Asn274Tyr |  |
| 319 | M | 1.8 | *EIF2B3* | c.562C>T | p.Gln188Tyr | c.1037T>C | p.Ile346Thr |  |
| 320 | F | 2.0 | *EIF2B3* | c.562C>T | p.Gln188Tyr | c.1037T>C | p.Ile346Thr |  |
| 321 | M | 1.2 | *EIF2B2* | c.254T>A | p.Val85Glu | c.976T>C | p.Ser326Pro |  |
| 322 | F | 2.0 | *EIF2B5* | c.235A>C | p.Thr79Pro | c.1004G>C | p.Cys335Ser |  |
| 323 | M | 2.3 | *EIF2B2* | c.254T>A | p.Val85Glu | c.911_913del | p.305del |  |
| 324 | M | 0.7 | *EIF2B5* | c.806G>A | p.Arg269Gln | c.1340C>T | p.Ser447Leu |  |
| 325 | F | 3.7 | *EIF2B5* | c.235A>C | p.Thr79Pro | c.915G>A | p.Met305Ile |  |
| 326 | M | 4.0 | *EIF2B2* | c.254T>A | p.Val85Glu | c.817A>C | p.Lys273Gln |  |
| 327 | F | 3.3 | *EIF2B5* | c.545C>T | p.Thr182Met | c.1340C>T | p.Ser447Leu |  |
| 328 | F | 4.0 | *EIF2B5* | c.805C>G | p.Asp137His | c.409G>C | P.Arg269Gly |  |
| 329 | F | not yet at 4.4y | *EIF2B5* | c.805C>G | p.Asp137His | c.409G>C | P.Arg269Gly |  |
| 330 | M | 1.8 | *EIF2B2* | c.254T>A | p.Val85Glu | c.922G>A | P.Val308Met |  |
| 331 | M | 2.7 | *EIF2B2* | c.3G>T | p.Met1Ile | c.922G>A | P.Val308Met |  |
| 332 | M | 3.3 | *EIF2B4* | c.728C>T | p.Pro243Leu | c.728C>T | p.Pro243Leu |  |
| 333 | M | 2.5 | *EIF2B5* | c.1484A>G | p.Tyr495Cys | c.337C>T | p.Arg113Cys |  |
| 334 | M | 0.8 | *EIF2B5* | c.1484A>G | p.Tyr495Cys | c.944G>A | p.Arg315His |  |
| 335 | F | 1.8 | *EIF2B5* | c.1241G>A | p.Cys414Tyr | c.338G>A | p.Arg113His |  |
| 336 | M | 4.1 | *EIF2B3* | c.272G>A | p.Arg91His | c.28G>C | p.Val10Leu |  |
| 337 | F | 1.0 | *EIF2B5* | c.1016G>A | p.Arg339Gln | c.943C>T | p.Arg315Cys |  |
| 338 | M | 1.0 | *EIF2B4* | c.1195A>C | p.Lys399Gln | c.932T>C | p.Ile311Thr |  |
| 339 | F | 2.0 | *EIF2B5* | c.1484A>G | p.Tyr495Cys | c.1688G>A | p.Arg563Gln |  |
| 340 | F | 2.0 | *EIF2B5* | c1484A>G | p.Tyr495Cys | c.1688G>A | p.Arg563Gln |  |
| 341 | F | 1.0 | *EIF2B5* | c.407G>A | p.Arg136His | c.407G>A | p.Arg136His |  |

M, male; F, female; uk, unknown.

282-341 cases were diagnosed in our center(Peking University First Hospital).

**References**

丁乐, 郭虎, 李杨, 何燕, 梁超, 金波, 卢孝鹏, and 郑帼 (2016). 白质消融性白质脑病2例报告并文献复习. *临床儿科杂志* 34**,** 815-818.

李霓, 林楠, 卢强, 黄颜, and 崔丽英 (2018). 反复意识障碍伴抽搐发作4年余. *中国现代神经疾病杂志* 18**,** 630-634.

刘宇, 王芳, 叶高波, and 杨琳 (2016). 白质消融性脑白质病临床与基因分析. *中国妇幼健康研究* 27**,** 1181-1184.

马慧, and 林剑军 (2019). 婴儿型白质消融性白质脑病MRI表现一例. *影像诊断与介入放射学* 28**,** 230-231.

莫庭庭, 李霞, 汪东, 王治静, 李东景, 梁丽丽, and 窦香君 (2020). 1例 EIF2B5新发突变所致儿童白质消融性脑病. *国际遗传学杂志* 43**,** 192-196.

徐化凤, 管红梅, 郭虎, 金波, 张新荣, and 高修成 (2019). 儿童白质消融性白质脑病的临床和MRI表现. *中国医学计算机成像杂志* 25**,** 559-563.

徐慧, 刘开运, 贾婉舒, 于佳会, and 姜采荣 (2019). 白质消融性白质脑病1例临床特征与基因类型分析. *临床儿科杂志* 37**,** 824-826,832.

张晓莉, 王丽君, 贾天明, 韩瑞, and 赵鑫 (2017). 先天型白质消融性白质脑病1例及其基因突变分析. *临床儿科杂志* 35**,** 806-809.

Accogli, A., Brais, B., Tampieri, D., and La Piana, R. (2019). Long-Standing Psychiatric Features as the Only Clinical Presentation of Vanishing White Matter Disease. *J Neuropsychiatry Clin Neurosci* 31**,** 276-279.

Alamri, H., Al Mutairi, F., Alothman, J., Alothaim, A., Alfadhel, M., and Alfares, A. (2016). Diabetic ketoacidosis in vanishing white matter. *Clin Case Rep* 4**,** 717-720.

Alías Hernández, I., Ramos Lizana, J., Aguirre Rodríguez, J., Aguilera López, P., Garzón Cabrera, M.I., and Entrala Bernal, C. (2013). [Left hemiparesis as a sign of onset of vanishing white matter disease. Identification of a new mutation]. *An Pediatr (Barc)* 79**,** 46-49.

Alsalem, A., Shaheen, R., and Alkuraya, F.S. (2012). Vanishing white matter disease caused by EIF2B2 mutation with the presentation of an adrenoleukodystrophy phenotype. *Gene* 496**,** 141-143.

Barros, S.R., Parreira, S.C.R., Miranda, A.F.B., Pereira, A.M.B., and Campos, N.M.P. (2018). New Insights in Vanishing White Matter Disease: Isolated Bilateral Optic Neuropathy in Adult Onset Disease. *J Neuroophthalmol* 38**,** 42-46.

Bektas, G., Yesil, G., Ozkan, M.U., Yildiz, E.P., Uzunhan, T.A., and Caliskan, M. (2018). Vanishing white matter disease with a novel EIF2B5 mutation: A 10-year follow-up. *Clin Neurol Neurosurg* 171**,** 190-193.

Buggle, F., Ciric, E., Boujan, T., Ohlenbusch, A., Gärtner, J., and Grau, A.J. (2019). [Vanishing white matter disease in adulthood]. *Nervenarzt* 90**,** 840-842.

Bursle, C., Yiu, E.M., Yeung, A., Freeman, J.L., Stutterd, C., Leventer, R.J., Vanderver, A., and Yaplito-Lee, J. (2020). Hyperinsulinaemic hypoglycaemia: A rare association of vanishing white matter disease. *JIMD Rep* 51**,** 11-16.

Carra-Dalliere, C., Horzinski, L., Ayrignac, X., Vukusic, S., Rodriguez, D., Mauguiere, F., Peter, L., Goizet, C., Bouhour, F., Denier, C., Confavreux, C., Obadia, M., Blanc, F., De Seze, J., Sedel, F., Guennoc, A.M., Sartori, E., Laplaud, D., Antoine, J.C., Fogli, A., Boespflug-Tanguy, O., and Labauge, P. (2011). [Natural history of adult-onset eIF2B-related disorders: a multicentric survey of 24 cases]. *Rev Neurol (Paris)* 167**,** 802-811.

Cohen, L., Manín, A., Medina, N., Rodríguez-Quiroga, S., González-Morón, D., Rosales, J., Amartino, H., Specola, N., Córdoba, M., Kauffman, M., and Vega, P. (2020). Argentinian clinical genomics in a leukodystrophies and genetic leukoencephalopathies cohort: Diagnostic yield in our first 9 years. *Ann Hum Genet* 84**,** 11-28.

Damásio, J., Van Der Lei, H.D., Van Der Knaap, M.S., and Santos, E. (2012). Late onset vanishing white matter disease presenting with learning difficulties. *J Neurol Sci* 314**,** 169-170.

Damon-Perriere, N., Menegon, P., Olivier, A., Boespflug-Tanguy, O., Niel, F., Creveaux, I., Dousset, V., Brochet, B., and Goizet, C. (2008). Intra-familial phenotypic heterogeneity in adult onset vanishing white matter disease. *Clin Neurol Neurosurg* 110**,** 1068-1071.

Denier, C., Orgibet, A., Roffi, F., Jouvent, E., Buhl, C., Niel, F., Boespflug-Tanguy, O., Said, G., and Ducreux, D. (2007). Adult-onset vanishing white matter leukoencephalopathy presenting as psychosis. *Neurology* 68**,** 1538-1539.

Ding, X.Q., Bley, A., Ohlenbusch, A., Kohlschütter, A., Fiehler, J., Zhu, W., and Lanfermann, H. (2012). Imaging evidence of early brain tissue degeneration in patients with vanishing white matter disease: a multimodal MR study. *J Magn Reson Imaging* 35**,** 926-932.

Dreha-Kulaczewski, S.F., Dechent, P., Finsterbusch, J., Brockmann, K., Gärtner, J., Frahm, J., and Hanefeld, F.A. (2008). Early reduction of total N-acetyl-aspartate-compounds in patients with classical vanishing white matter disease. A long-term follow-up MRS study. *Pediatr Res* 63**,** 444-449.

Esmer, C., Blanco Hernández, G., Saavedra Alanís, V., Reyes Vaca, J.G., and Bravo Oro, A. (2017). [Association between homozygous c.318A>GT mutation in exon 2 of the EIF2B5 gene and the infantile form of vanishing white matter leukoencephalopathy]. *Bol Med Hosp Infant Mex* 74**,** 364-369.

Federico, A., Scali, O., Stromillo, M.L., Di Perri, C., Bianchi, S., Sicurelli, F., De Stefano, N., Malandrini, A., and Dotti, M.T. (2006). Peripheral neuropathy in vanishing white matter disease with a novel EIF2B5 mutation. *Neurology* 67**,** 353-355.

Fogli, A., Dionisi-Vici, C., Deodato, F., Bartuli, A., Boespflug-Tanguy, O., and Bertini, E. (2002a). A severe variant of childhood ataxia with central hypomyelination/vanishing white matter leukoencephalopathy related to EIF21B5 mutation. *Neurology* 59**,** 1966-1968.

Fogli, A., Schiffmann, R., Bertini, E., Ughetto, S., Combes, P., Eymard-Pierre, E., Kaneski, C.R., Pineda, M., Troncoso, M., Uziel, G., Surtees, R., Pugin, D., Chaunu, M.P., Rodriguez, D., and Boespflug-Tanguy, O. (2004). The effect of genotype on the natural history of eIF2B-related leukodystrophies. *Neurology* 62**,** 1509-1517.

Fogli, A., Wong, K., Eymard-Pierre, E., Wenger, J., Bouffard, J.P., Goldin, E., Black, D.N., Boespflug-Tanguy, O., and Schiffmann, R. (2002b). Cree leukoencephalopathy and CACH/VWM disease are allelic at the EIF2B5 locus. *Ann Neurol* 52**,** 506-510.

Fontenelle, L.M., Scheper, G.C., Brandão, L., and Van Der Knaap, M.S. (2008). Atypical presentation of vanishing white matter disease. *Arq Neuropsiquiatr* 66**,** 549-551.

Gascon-Bayarri, J., Campdelacreu, J., Sánchez-Castañeda, C., Martínez-Yélamos, S., Moragas, M., Scheper, G.C., Van Der Knaap, M.S., and Reñé, R. (2009). Leukoencephalopathy with vanishing white matter presenting with presenile dementia. *J Neurol Neurosurg Psychiatry* 80**,** 810-811.

Ghezzi, L., Scarpini, E., Rango, M., Arighi, A., Bassi, M.T., Tenderini, E., De Riz, M., Jacini, F., Fumagalli, G.G., Pietroboni, A.M., Galimberti, D., and Bresolin, N. (2012). A 66-year-old patient with vanishing white matter disease due to the p.Ala87Val EIF2B3 mutation. *Neurology* 79**,** 2077-2078.

Gowda, V.K., Srinivasan, V.M., Bhat, M., and Benakappa, A. (2017). Case of Childhood Ataxia with Central Nervous System Hypomyelination with a Novel Mutation in EIF2B3 gene. *J Pediatr Neurosci* 12**,** 196-198.

Gungor, O., Ozkaya, A.K., Hirfanoglu, T., Dilber, C., and Aydin, K. (2015). A rare mutation in EIF2B4 gene in an epileptic child with vanishing white matter disease: a case report. *Genet Couns* 26**,** 41-46.

Harder, S., Gourgaris, A., Frangou, E., Hopp, K., Huntsman, R., Lowry, N., Seshia, S., Lemire, E., Robinson, C., and Tynan, J. (2010). Clinical and neuroimaging findings of Cree leukodystrophy: a retrospective case series. *AJNR Am J Neuroradiol* 31**,** 1418-1423.

Hata, Y., Kinoshita, K., Miya, K., Hirono, K., Ichida, F., Yoshida, K., and Nishida, N. (2014). An autopsy case of infantile-onset vanishing white matter disease related to an EIF2B2 mutation (V85E) in a hemizygous region. *Int J Clin Exp Pathol* 7**,** 3355-3362.

Herwerth, M., Schwaiger, B.J., Kreiser, K., Hemmer, B., and Ilg, R. (2015). Adult-onset vanishing white matter disease as differential diagnosis of primary progressive multiple sclerosis: a case report. *Mult Scler* 21**,** 666-668.

Hettiaracchchi, D., Neththikumara, N., Pathirana, B., Padeniya, A., and Dissanayake, V.H.W. (2018). A Novel Mutation in the EIF2B4 Gene Associated with Leukoencephalopathy with Vanishing White Matter. *Case Rep Pediatr* 2018**,** 2731039.

Horzinski, L., Gonthier, C., Rodriguez, D., Scherer, C., Boespflug-Tanguy, O., and Fogli, A. (2008). Exon deletion in the non-catalytic domain of eIF2Bepsilon due to a splice site mutation leads to infantile forms of CACH/VWM with severe decrease of eIF2B GEF activity. *Ann Hum Genet* 72**,** 410-415.

Horzinski, L., Huyghe, A., Cardoso, M.C., Gonthier, C., Ouchchane, L., Schiffmann, R., Blanc, P., Boespflug-Tanguy, O., and Fogli, A. (2009). Eukaryotic initiation factor 2B (eIF2B) GEF activity as a diagnostic tool for EIF2B-related disorders. *PLoS One* 4**,** e8318.

Huntsman, R.J., Seshia, S., Lowry, N., Lemire, E.G., and Harder, S.L. (2007). Peripheral neuropathy in a child with Cree leukodystrophy. *J Child Neurol* 22**,** 766-768.

Hyun, S.E., Choi, B.S., Jang, J.H., Jeon, I., Jang, D.H., and Ryu, J.S. (2019). Correlation Between Vanishing White Matter Disease and Novel Heterozygous EIF2B3 Variants Using Next-Generation Sequencing: A Case Report. *Ann Rehabil Med* 43**,** 234-238.

Imam, I., Brown, J., Lee, P., Thomas, P.K., and Manji, H. (2011). Ovarioleukodystrophy: report of a case with the c.338G>A (p.Arg113His) mutation on exon 3 and the c.896G>A (p.Arg299His) mutation on exon 7 of the EIF2B5 gene. *BMJ Case Rep* 2011.

Jansen, A.C., Andermann, E., Niel, F., Creveaux, I., Boespflug-Tanguy, O., and Andermann, F. (2008). Leucoencephalopathy with vanishing white matter may cause progressive myoclonus epilepsy. *Epilepsia* 49**,** 910-913.

Jurkiewicz, E., Mierzewska, H., Bekiesińska-Figatowska, M., Pakua-Kościesza, I., Kmieć, T., Scheper, G., Van Der Knaap, M.S., and Pronicka, E. (2005). MRI of a family with leukoencephalypathy with vanishing white matter. *Pediatr Radiol* 35**,** 1027-1030.

Kaczorowska, M., Kuczynski, D., Jurkiewicz, E., Scheper, G.C., Van Der Knaap, M.S., and Jozwiak, S. (2006). Acute fright induces onset of symptoms in vanishing white matter disease-case report. *Eur J Paediatr Neurol* 10**,** 192-193.

Klingelhoefer, L., Misbahuddin, A., Jawad, T., Mellers, J., Jarosz, J., Weeks, R., and Ray Chaudhuri, K. (2014). Vanishing white matter disease presenting as opsoclonus myoclonus syndrome in childhood--a case report and review of the literature. *Pediatr Neurol* 51**,** 157-164.

Labauge, P., Horzinski, L., Ayrignac, X., Blanc, P., Vukusic, S., Rodriguez, D., Mauguiere, F., Peter, L., Goizet, C., Bouhour, F., Denier, C., Confavreux, C., Obadia, M., Blanc, F., De Sèze, J., Fogli, A., and Boespflug-Tanguy, O. (2009). Natural history of adult-onset eIF2B-related disorders: a multi-centric survey of 16 cases. *Brain* 132**,** 2161-2169.

Lee, H.N., Koh, S.H., Lee, K.Y., Ki, C.S., and Lee, Y.J. (2009). Late-onset vanishing white matter disease with compound heterozygous EIF2B5 gene mutations. *Eur J Neurol* 16**,** e42-43.

Lee, J.S., Lee, S., Choi, M., Lim, B.C., Choi, J., Kim, K.J., Cheon, J.E., Kim, I.O., and Chae, J.H. (2017). eIF2B-related multisystem disorder in two sisters with atypical presentations. *Eur J Paediatr Neurol* 21**,** 404-409.

Lucas, M., Suarez, R., Marcos, A., Solano, F., Venegas, A., Garcia-Sanchez, M.I., Ortiz, L., and Izquierdo, G. (2007). Arg113His mutation of vanishing white matter is not present in multiple sclerosis. *Mult Scler* 13**,** 424-427.

Mascalchi, M., De Grandis, D., Ginestroni, A., Pratesi, A., Della Nave, R., Scheper, G.C., and Van Der Knaap, M.S. (2006). Early MR imaging and spectroscopy appearance of eIF2B-related leukoencephalopathy. *Neurology* 67**,** 537-538.

Mathis, S., Scheper, G.C., Baumann, N., Petit, E., Gil, R., Van Der Knaap, M.S., and Neau, J.P. (2008). The ovarioleukodystrophy. *Clin Neurol Neurosurg* 110**,** 1035-1037.

Matsui, M., Mizutani, K., Ohtake, H., Miki, Y., Ishizu, K., Fukuyama, H., Shimohata, T., Onodera, O., Nishizawa, M., Takayama, Y., and Shibasaki, H. (2007). Novel mutation in EIF2B gene in a case of adult-onset leukoencephalopathy with vanishing white matter. *Eur Neurol* 57**,** 57-58.

Matsukawa, T., Wang, X., Liu, R., Wortham, N.C., Onuki, Y., Kubota, A., Hida, A., Kowa, H., Fukuda, Y., Ishiura, H., Mitsui, J., Takahashi, Y., Aoki, S., Takizawa, S., Shimizu, J., Goto, J., Proud, C.G., and Tsuji, S. (2011). Adult-onset leukoencephalopathies with vanishing white matter with novel missense mutations in EIF2B2, EIF2B3, and EIF2B5. *Neurogenetics* 12**,** 259-261.

Mierzewska, H., Van Der Knaap, M.S., Scheper, G.C., Jurkiewicz, E., Schmidt-Sidor, B., and Szymańska, K. (2006). Leukoencephalopathy with vanishing white matter due to homozygous EIF2B2 gene mutation. First Polish cases. *Folia Neuropathol* 44**,** 144-148.

Ohlenbusch, A., Henneke, M., Brockmann, K., Goerg, M., Hanefeld, F., Kohlschütter, A., and Gärtner, J. (2005). Identification of ten novel mutations in patients with eIF2B-related disorders. *Hum Mutat* 25**,** 411.

Ohtake, H., Shimohata, T., Terajima, K., Kimura, T., Jo, R., Kaseda, R., Iizuka, O., Takano, M., Akaiwa, Y., Goto, H., Kobayashi, H., Sugai, T., Muratake, T., Hosoki, T., Shioiri, T., Okamoto, K., Onodera, O., Tanaka, K., Someya, T., Nakada, T., and Tsuji, S. (2004). Adult-onset leukoencephalopathy with vanishing white matter with a missense mutation in EIF2B5. *Neurology* 62**,** 1601-1603.

Pena, L.D.M., Jiang, Y.H., Schoch, K., Spillmann, R.C., Walley, N., Stong, N., Rapisardo Horn, S., Sullivan, J.A., Mcconkie-Rosell, A., Kansagra, S., Smith, E.C., El-Dairi, M., Bellet, J., Keels, M.A., Jasien, J., Kranz, P.G., Noel, R., Nagaraj, S.K., Lark, R.K., Wechsler, D.S.G., Del Gaudio, D., Leung, M.L., Hendon, L.G., Parker, C.C., Jones, K.L., Goldstein, D.B., and Shashi, V. (2018). Looking beyond the exome: a phenotype-first approach to molecular diagnostic resolution in rare and undiagnosed diseases. *Genet Med* 20**,** 464-469.

Pineda, M., A, R.P., Baquero, M., O'callaghan, M., Aracil, A., Van Der Knaap, M., and Scheper, G.C. (2008). Vanishing white matter disease associated with progressive macrocephaly. *Neuropediatrics* 39**,** 29-32.

Porciuncula, R., Spada, P., and Goulart, K.O.B. (2018). LEUKOENCEPHALOPATHY WITH EVANESCENT WHITE MATTER: A CASE REPORT. *Rev Paul Pediatr* 36**,** 515-518.

Prange, H., and Weber, T. (2011). [Vanishing white matter disease: a stress-related leukodystrophy]. *Nervenarzt* 82**,** 1330-1334.

Ramaswamy, V., Chan, A.K., and Kolski, H.K. (2006). Vanishing white matter disease with periodic (paroxysmal) hemiparesis. *Pediatr Neurol* 35**,** 65-68.

Riecker, A., Nägele, T., Henneke, M., and Schöls, L. (2007). Late onset vanishing white matter disease. *J Neurol* 254**,** 544-545.

Robbins, K., Arraj, P., Dengle Sanchez, L., Godiyal, N., Veltkamp, D.L., and Pfeifer, C.M. (2021). CT and MRI findings in infantile vanishing white matter. *Radiol Case Rep* 16**,** 116-118.

Robinson, M., Rossignol, E., Brais, B., Rouleau, G., Arbour, J.F., and Bernard, G. (2014). Vanishing white matter disease in French-Canadian patients from Quebec. *Pediatr Neurol* 51**,** 225-232.

Sambati, L., Agati, R., Bacci, A., Bianchi, S., and Capellari, S. (2013). Vanishing white matter disease: an Italian case with A638G mutation in exon 5 of EIF2B2 gene, an unusual early onset and a long course. *Neurol Sci* 34**,** 1235-1238.

Sharma, S., Ajij, M., Singh, V., and Aneja, S. (2015). Vanishing white matter disease with mutations in EIF2B5 gene. *Indian J Pediatr* 82**,** 93-95.

Sharma, S., Arya, R., Raju, K.N., Kumar, A., Scheper, G.C., Van Der Knaap, M.S., and Gulati, S. (2011). Vanishing white matter disease associated with ptosis and myoclonic seizures. *J Child Neurol* 26**,** 366-368.

Shimada, S., Shimojima, K., Sangu, N., Hoshino, A., Hachiya, Y., Ohto, T., Hashi, Y., Nishida, K., Mitani, M., Kinjo, S., Tsurusaki, Y., Matsumoto, N., Morimoto, M., and Yamamoto, T. (2015). Mutations in the genes encoding eukaryotic translation initiation factor 2B in Japanese patients with vanishing white matter disease. *Brain Dev* 37**,** 960-966.

Singh, R.R., Livingston, J., Lim, M., Berry, I.R., and Siddiqui, A. (2017). An unusual neuroimaging finding and response to immunotherapy in a child with genetically confirmed vanishing white matter disease. *Eur J Paediatr Neurol* 21**,** 410-413.

Song, H., Haeri, S., Vogel, H., Van Der Knaap, M., and Van Haren, K. (2017). Postmortem Whole Exome Sequencing Identifies Novel EIF2B3 Mutation With Prenatal Phenotype in 2 Siblings. *J Child Neurol* 32**,** 867-870.

Takano, K., Tsuyusaki, Y., Sato, M., Takagi, M., Anzai, R., Okuda, M., Iai, M., Yamashita, S., Okabe, T., Aida, N., Tsurusaki, Y., Saitsu, H., Matsumoto, N., and Osaka, H. (2015). A Japanese girl with an early-infantile onset vanishing white matter disease resembling Cree leukoencephalopathy. *Brain Dev* 37**,** 638-642.

Trimouille, A., Marguet, F., Sauvestre, F., Lasseaux, E., Pelluard, F., Martin-Négrier, M.L., Plaisant, C., Rooryck, C., Lacombe, D., Arveiler, B., Boespflug-Tanguy, O., Naudion, S., and Laquerrière, A. (2020). Foetal onset of EIF2B related disorder in two siblings: cerebellar hypoplasia with absent Bergmann glia and severe hypomyelination. *Acta Neuropathol Commun* 8**,** 48.

Turón-Viñas, E., Pineda, M., Cusí, V., López-Laso, E., Del Pozo, R.L., Gutiérrez-Solana, L.G., Moreno, D.C., Sierra-Córcoles, C., Olabarrieta-Hoyos, N., Madruga-Garrido, M., Aguirre-Rodríguez, J., González-Álvarez, V., O'callaghan, M., Muchart, J., and Armstrong-Moron, J. (2014). Vanishing white matter disease in a spanish population. *J Cent Nerv Syst Dis* 6**,** 59-68.

Unal, O., Ozgen, B., Orhan, D., Tokatli, A., Hismi, B.O., Dursun, A., Coskun, T., and Kalkanoglu-Sivri, H.S. (2013). Vanishing White Matter With Hepatomegaly and Hypertriglyceridemia Attacks. *J Child Neurol* 28**,** 1509-1512.

Valálik, I., Van Der Knaap, M.S., Scheper, G.C., Jobbágy, A., Liptai, Z., and Csókay, A. (2012). Long-term tremor control with bilateral Vim-DBS in vanishing white matter disease. *Parkinsonism Relat Disord* 18**,** 1048-1050.

Van Der Knaap, M.S., Leegwater, P.A., Van Berkel, C.G., Brenner, C., Storey, E., Di Rocco, M., Salvi, F., and Pronk, J.C. (2004). Arg113His mutation in eIF2Bepsilon as cause of leukoencephalopathy in adults. *Neurology* 62**,** 1598-1600.

Van Der Knaap, M.S., Van Berkel, C.G., Herms, J., Van Coster, R., Baethmann, M., Naidu, S., Boltshauser, E., Willemsen, M.A., Plecko, B., Hoffmann, G.F., Proud, C.G., Scheper, G.C., and Pronk, J.C. (2003). eIF2B-related disorders: antenatal onset and involvement of multiple organs. *Am J Hum Genet* 73**,** 1199-1207.

Van Der Lei, H.D., Steenweg, M.E., Barkhof, F., De Grauw, T., D'hooghe, M., Morton, R., Shah, S., Wolf, N., and Van Der Knaap, M.S. (2012). Characteristics of early MRI in children and adolescents with vanishing white matter. *Neuropediatrics* 43**,** 22-26.

Van Diemen, C.C., Kerstjens-Frederikse, W.S., Bergman, K.A., De Koning, T.J., Sikkema-Raddatz, B., Van Der Velde, J.K., Abbott, K.M., Herkert, J.C., Löhner, K., Rump, P., Meems-Veldhuis, M.T., Neerincx, P.B.T., Jongbloed, J.D.H., Van Ravenswaaij-Arts, C.M., Swertz, M.A., Sinke, R.J., Van Langen, I.M., and Wijmenga, C. (2017). Rapid Targeted Genomics in Critically Ill Newborns. *Pediatrics* 140.

Vermeulen, G., Seidl, R., Mercimek-Mahmutoglu, S., Rotteveel, J.J., Scheper, G.C., and Van Der Knaap, M.S. (2005). Fright is a provoking factor in vanishing white matter disease. *Ann Neurol* 57**,** 560-563.

Villar-Quiles, R.N., Delgado-Suarez, C., Jorquera-Moya, M., Arpa-Gutierrez, J., and Ortega-Suero, G. (2018). Teaching NeuroImages: Adult-onset vanishing white matter disease. *Neurology* 90**,** e1091-e1092.

Vinogradsky, E.I., and Otallah, S.I. (2019). Vanishing White Matter Disease Diagnosis After Athletic Concussion in an Adolescent Male Patient. *Clin J Sport Med*.

Wang, X., He, F., Yin, F., Chen, C., Wu, L., Yang, L., and Peng, J. (2016). The use of targeted genomic capture and massively parallel sequencing in diagnosis of Chinese Leukoencephalopathies. *Sci Rep* 6**,** 35936.

Wilson, C.J., Pronk, J.C., and Van Der Knaap, M.S. (2005). Vanishing white matter disease in a child presenting with ataxia. *J Paediatr Child Health* 41**,** 65-67.

Wong, S.S., Luk, D.C., Wong, V.C., Scheper, G.C., and Van Der Knaap, M.S. (2008). Vanishing white matter disease: the first reported chinese patient. *J Child Neurol* 23**,** 710-714.

Yavuz, H. (2017). A Review of Infantile Vanishing White Matter Disease and A New Mutation. *Acta Neurol Taiwan* 26**,** 167-176.
