# Supplementary material for: Correlation Between Genotype and Age of Onset in Leukoencephalopathy With Vanishing White Matter
Source: Front Genet. 2021 Oct 20;12:729777. doi: 10.3389/fgene.2021.729777 (PMC8564072; doi:10.3389/fgene.2021.729777)
Supplement: Supplementary file 2 [file Table2.DOCX]

Supplementary Table 2. 154 missense mutations in 303 VWM patients

| *EIF2B* gene | Number | DNA mutation | Protein change | Affected domain |
| --- | --- | --- | --- | --- |
| *EIF2B1* | 1 | c.146T>G | p.Leu49Arg | regulatory domain |
| *EIF2B1* | 2 | c.715T>G | p.Phe239Val | regulatory domain |
| *EIF2B1* | 3 | c.547G>T | p.Val183Phe | regulatory domain |
| *EIF2B1* | 4 | c.328A>G | p.Lys110Glu | regulatory domain |
| *EIF2B2* | 5 | c.254T>A | p.Val85Glu | regulatory domain |
| *EIF2B2* | 6 | c.677T>A | p.Met226Lys | regulatory domain |
| *EIF2B2* | 7 | c.682A>G | p.Arg228Gly | regulatory domain |
| *EIF2B2* | 8 | c.638A>G | p.Glu213Gly | regulatory domain |
| *EIF2B2* | 9 | c.817A>C | p.Lys273Gln | regulatory domain |
| *EIF2B2* | 10 | c.599G>T | p.Gly200Val | regulatory domain |
| *EIF2B2* | 11 | c.880G>T | p.Val294Phe | regulatory domain |
| *EIF2B2* | 12 | c.512C>T | p.Ser171Phe | regulatory domain |
| *EIF2B2* | 13 | c.803G>A | p.Cys268Tyr | regulatory domain |
| *EIF2B2* | 14 | c.818A>G | p.Lys273His | regulatory domain |
| *EIF2B2* | 15 | c.599G>C | p.Gly200Ala | regulatory domain |
| *EIF2B2* | 16 | c.871C>T | p.Pro291Ser | regulatory domain |
| *EIF2B2* | 17 | c.233T>A | p.Val78Glu | regulatory domain |
| *EIF2B2* | 18 | c.922G>A | p.Val308Met | regulatory domain |
| *EIF2B2* | 19 | c.995C>T | p.Ala332Val | regulatory domain |
| *EIF2B2* | 20 | c.976T>C | p.Ser326Pro | regulatory domain |
| *EIF2B2* | 21 | c.3G>T | p.Met1Ile | other homologous domain |
| *EIF2B3* | 22 | c.260C>T | p.Ala87Val | NT domain |
| *EIF2B3* | 23 | c.706C>G | p.Gln236Glu | other homologous domain |
| *EIF2B3* | 24 | c.89T>C | p.Val30Ala | NT domain |
| *EIF2B3* | 25 | c.97A>G | p.Lys33Glu | NT domain |
| *EIF2B3* | 26 | c.1270T>G | p.Cys424Gly | I-patch domain |
| *EIF2B3* | 27 | c.136G>A | p.Val46Ile | NT domain |
| *EIF2B3* | 28 | c.272G>A | p.Arg91His | NT domain |
| *EIF2B3* | 29 | c.144T>A | p.Phe48Leu | NT domain |
| *EIF2B3* | 30 | c.80T>A | p.Leu27Gln | NT domain |
| *EIF2B3* | 31 | c.604G>A | p.Ala202Thr | other homologous domain |
| *EIF2B3* | 32 | c.41C>T | p.Ser14Phe | NT domain |
| *EIF2B3* | 33 | NA | p.Val30Gly | NT domain |
| *EIF2B3* | 34 | NA | p.Arg226Gln | other homologous domain |
| *EIF2B3* | 35 | c.674G>A | p.Arg225Gln | other homologous domain |
| *EIF2B3* | 36 | c.1023T>G | p.His341Gln | I-patch domain |
| *EIF2B3* | 37 | c.407A>C | p.Gln136Pro | other homologous domain |
| *EIF2B3* | 38 | c.140G>A | p.Gly47Glu | NT domain |
| *EIF2B3* | 39 | c.1037T>C | p.Ile346Thr | I-patch domain |
| *EIF2B3* | 40 | c.935G>A | p.Arg312Gln | other homologous domain |
| *EIF2B3* | 41 | c.32G>T | p.Gly11Val | NT domain |
| *EIF2B3* | 42 | c.965C>G | p.Ala322Gly | other homologous domain |
| *EIF2B3* | 43 | c.562C>T | p.Gln188Tyr | other homologous domain |
| *EIF2B3* | 44 | c.28G>C | p.Val10Leu | NT domain |
| *EIF2B4* | 45 | c.725C>T | p.Pro242Leu | regulatory domain |
| *EIF2B4* | 46 | c.1301T>C | p.Leu434Pro | regulatory domain |
| *EIF2B4* | 47 | c.628G>T | p.Gly210Cys | regulatory domain |
| *EIF2B4* | 48 | c.626G>A | p.Arg209Gln | regulatory domain |
| *EIF2B4* | 49 | c.1298​​C>T | p.Pro433Leu | regulatory domain |
| *EIF2B4* | 50 | c.614C>T | p.Pro205Leu | regulatory domain |
| *EIF2B4* | 51 | c.1334G>A | p.Arg445His | regulatory domain |
| *EIF2B4* | 52 | c.556T>A | p.Tyr186Asn | regulatory domain |
| *EIF2B4* | 53 | c.1070G>A | p.Arg357Gln | regulatory domain |
| *EIF2B4* | 54 | c.1091G>A | p.Arg364Gln | regulatory domain |
| *EIF2B4* | 55 | c.1090C>T | p.Arg364Trp | regulatory domain |
| *EIF2B4* | 56 | c.1120C>T | p.Arg374Cys | regulatory domain |
| *EIF2B4* | 57 | c.818T>C | p.Met273Thr | regulatory domain |
| *EIF2B4* | 58 | c.1346C>T | p.Thr499Ile | regulatory domain |
| *EIF2B4* | 59 | c.1069C>T | p.Arg357Trp | regulatory domain |
| *EIF2B4* | 60 | c.728C>T | p.Pro243Leu | regulatory domain |
| *EIF2B4* | 61 | c.1465T>C | p.Tyr489His | regulatory domain |
| *EIF2B4* | 62 | c.1447C>T | p.Arg483Trp | regulatory domain |
| *EIF2B4* | 63 | c.1172C>A | p.Ala391Asp | regulatory domain |
| *EIF2B4* | 64 | c.1180C>T | p.Leu394phe | regulatory domain |
| *EIF2B4* | 65 | c.407A>G | p.Gln136Arg | other homologous domain |
| *EIF2B4* | 66 | c.691G>A | p.Gly231Ser | regulatory domain |
| *EIF2B4* | 67 | c.1459C>T | p.Arg487Trp | regulatory domain |
| *EIF2B4* | 68 | c.1382A>G | p.Tyr461Cys | regulatory domain |
| *EIF2B4* | 69 | c.1565C>T | p.Thr522Met | regulatory domain |
| *EIF2B4* | 70 | c.728C>T | p.Pro243Leu | regulatory domain |
| *EIF2B4* | 71 | c.1195A>C | p.Lys399Gln | regulatory domain |
| *EIF2B4* | 72 | c.932T>C | p.Ile311Thr | regulatory domain |
| *EIF2B5* | 73 | c.584G>A | p.Arg195His | other homologous domain |
| *EIF2B5* | 74 | c.436T>C | p. Ser146Pro | NT domain |
| *EIF2B5* | 75 | c.468C>G | p.Ile156Met | NT domain |
| *EIF2B5* | 76 | c.1165G>A | p.Val389Met | I-patch domain |
| *EIF2B5* | 77 | c.338G>A | p.Arg113His | NT domain |
| *EIF2B5* | 78 | c.896G>A | p.Arg299His | other homologous domain |
| *EIF2B5* | 79 | c.1688G>A | p.Arg563Gln | catalytic domain |
| *EIF2B5* | 80 | c.806G>A | p.Arg269Gln | other homologous domain |
| *EIF2B5* | 81 | c.956A>G | p.Tyr319Cys | other homologous domain |
| *EIF2B5* | 82 | c.1015C>T | p.Arg339Trp | other homologous domain |
| *EIF2B5* | 83 | c.1208C>T | p.Ala403Val | I-patch domain |
| *EIF2B5* | 84 | c.241G>A | p.Glu81Lys | NT domain |
| *EIF2B5* | 85 | c.203T>C | p.Leu68Ser | NT domain |
| *EIF2B5* | 86 | c.318A>T | p.Leu106Phe | NT domain |
| *EIF2B5* | 87 | c.1004G>C | p.Cys335Ser | other homologous domain |
| *EIF2B5* | 88 | c.1484A>G | p.Tyr495Cys | other homologous domain |
| *EIF2B5* | 89 | c.1223T>c | p.Ile408Thr | I-patch domain |
| *EIF2B5* | 90 | c.915G>A | p.Met305Ile | other homologous domain |
| *EIF2B5* | 91 | c.1154T>C | p.Ile385Thr | I-patch domain |
| *EIF2B5* | 92 | c.230A>G | p.Asp77Gly | NT domain |
| *EIF2B5* | 93 | c.407G>A | p.Arg136His | NT domain |
| *EIF2B5* | 94 | c.314A>G | p.His105Arg | NT domain |
| *EIF2B5* | 95 | c.406C>T | p.Arg136Cys | NT domain |
| *EIF2B5* | 96 | c.943C>T | p.Arg315Cys | other homologous domain |
| *EIF2B5* | 97 | c.395G>C | p.Gly132Ala | NT domain |
| *EIF2B5* | 98 | c.475A>G | p.Ile159Val | NT domain |
| *EIF2B5* | 99 | c.5C>T | p.Ala2Val | other homologous domain |
| *EIF2B5* | 100 | c.631A>G | p.Arg211Gly | other homologous domain |
| *EIF2B5* | 101 | c.1946T>C | p.Ile649Thr | catalytic domain |
| *EIF2B5* | 102 | c.449T>G | p.Leu150Arg | NT domain |
| *EIF2B5* | 103 | c.1355A>G | p.His452Arg | other homologous domain |
| *EIF2B5* | 104 | c.626G>A | p.Arg209Gln | other homologous domain |
| *EIF2B5* | 105 | c.1399C>T | p.Arg467Trp | other homologous domain |
| *EIF2B5* | 106 | c.808G>C | p.Asp270His | other homologous domain |
| *EIF2B5* | 107 | c.743A>T | p.His248Leu | other homologous domain |
| *EIF2B5* | 108 | c.1448A>G | p.Tyr483Cys | other homologous domain |
| *EIF2B5* | 109 | c.641A>G | p.His214Arg | other homologous domain |
| *EIF2B5* | 110 | c.664C>T | p.Arg222Trp | other homologous domain |
| *EIF2B5* | 111 | c.1241G>A | p.Cys414Tyr | I-patch domain |
| *EIF2B5* | 112 | c.1340C>T | p.Ser447Leu | other homologous domain |
| *EIF2B5* | 113 | c.545C>T | p.Thr182Met | other homologous domain |
| *EIF2B5* | 114 | NA | p.Pro87Leu | NT domain |
| *EIF2B5* | 115 | c.1280C>T | p.Pro427Leu | I-patch domain |
| *EIF2B5* | 116 | c.468G>C | p.Ile156Met | NT domain |
| *EIF2B5* | 117 | NA | p.Met608lle | catalytic domain |
| *EIF2B5* | 118 | c.1459G>A | p.Glu487Lys | other homologous domain |
| *EIF2B5* | 119 | c.1810C>T | p.Pro604Ser | catalytic domain |
| *EIF2B5* | 120 | c.271A>G | p.Thr91Ala | NT domain |
| *EIF2B5* | 121 | c.331T>C | p.Trp111Ala | NT domain |
| *EIF2B5* | 122 | c.1360C>T | p.Pro454Ser | other homologous domain |
| *EIF2B5* | 123 | c.929G>T | p.Cys310Phe | other homologous domain |
| *EIF2B5* | 124 | c.967C>T | p.Pro323Ser | other homologous domain |
| *EIF2B5* | 125 | c.166T>G | p.Phe56Val | NT domain |
| *EIF2B5* | 126 | c.944G>A | p.Arg315His | other homologous domain |
| *EIF2B5* | 127 | c.1274T>G | p.Leu425Arg | I-patch domain |
| *EIF2B5* | 128 | c.1160T>G | p.Asp387Gly | I-patch domain |
| *EIF2B5* | 129 | c.1028A>G | p.Tyr343Cys | other homologous domain |
| *EIF2B5* | 130 | c.1153A>G | p.Ile385Val | I-patch domain |
| *EIF2B5* | 131 | c.1948G>A | p.Glu650Leu | catalytic domain |
| *EIF2B5* | 132 | c.806G>T | p.Arg269Leu | other homologous domain |
| *EIF2B5* | 133 | c.592G>A | p.Glu198Lys | other homologous domain |
| *EIF2B5* | 134 | c.1016G>T | c.Arg339Pro | other homologous domain |
| *EIF2B5* | 135 | c.47C>A | p.Ala16Asp | other homologous domain |
| *EIF2B5* | 136 | c.161G>C | p.Arg54Pro | NT domain |
| *EIF2B5* | 137 | c.337C>T | p.Arg113Cys | NT domain |
| *EIF2B5* | 138 | c.218T>G | p.Val73Glu | NT domain |
| *EIF2B5* | 139 | c.583C>T | p.Arg195Cys | other homologous domain |
| *EIF2B5* | 140 | c.1289T>C | p.Val430Ala | I-patch domain |
| *EIF2B5* | 141 | c.925G>C | p.Val309Leu | other homologous domain |
| *EIF2B5* | 142 | c.1016G>A | p.Arg339Gln | other homologous domain |
| *EIF2B5* | 143 | c.232T>C | p.Tyr78His | NT domain |
| *EIF2B5* | 144 | c.536C>T | p.Ser179Phe | other homologous domain |
| *EIF2B5* | 145 | c.1126A>G | p.Asn376Asp | I-patch domain |
| *EIF2B5* | 146 | c.185A>T | p.Asp62Val | NT domain |
| *EIF2B5* | 147 | c.1016G>C | p.Arg339Pro | other homologous domain |
| *EIF2B5* | 148 | c.1157G>A | P.Gly386Val | I-patch domain |
| *EIF2B5* | 149 | c.337C>A | p.Arg113Cys | NT domain |
| *EIF2B5* | 150 | c.947G>A | p.Arg316Gln | other homologous domain |
| *EIF2B5* | 151 | c.1352T>C | p.Leu451Ser | other homologous domain |
| *EIF2B5* | 152 | c.235A>C | p.Thr79Pro | NT domain |
| *EIF2B5* | 153 | c.805C>G | p.Asp137His | NT domain |
| *EIF2B5* | 154 | c.409G>C | P.Arg269Gly | other homologous domain |

NA, not available.
